# Targeting iron regulatory protein 2 (IRP2) to disrupt iron metabolism enhances radiosensitivity through mitochondrial dysfunction in breast cancer cells

**DOI:** 10.1038/s41420-025-02653-z

**Published:** 2025-07-31

**Authors:** Ye Yeong Jeong, Jieon Hwang, Areum Park, Sungmin Cho, Inyoung Cho, Soseul Won, You Me Shin, Sung Eun Kim, Chan Hoon Maeng, Jaemoon Yang, Minhee Ku, Hyuk Lee, Sang Joon Shin

**Affiliations:** 1 Department of Medicine, Yonsei University College of Medicine, Seoul, 03722 Republic of Korea; 2 Songdang Institute for Cancer Research, Yonsei University College of Medicine, Seoul, 03722 Republic of Korea; 3 Infectious Diseases Therapeutic Research Center, Korea Research Institute of Chemical Technology, Daejeon, 34114 Republic of Korea; 4 Department of Chemistry, Yonsei University, Seoul, 03722 Republic of Korea; 5 Department of Clinical Drug Discovery and Development, Yonsei University College of Medicine, Seoul, Republic of Korea; 6 Department of Radiology, College of Medicine, Yonsei University, Seoul, 03722 Republic of Korea; 7 Convergence Research Center for Systems Molecular Radiological Science, Yonsei University, Seoul, 03722 Republic of Korea; 8 Division of Medical Oncology, Department of Internal Medicine, Yonsei Cancer Center, Yonsei University College of Medicine, Seoul, 03722 Republic of Korea

**Keywords:** Breast cancer, Cancer therapeutic resistance

## Abstract

Iron regulatory protein (IRP2) plays a key role in regulating iron metabolism and enables cell survival by activating mitochondrial function. Targeting IRP2 to disrupt iron homeostasis is a promising strategy for enhancing the efficacy of cancer treatments. Depletion of IRP2 in breast cancer (BC) cells is associated with sensitivity to radiation therapy (RT), and inhibition of IRP2 prior to RT significantly reduces cell viability compared with radiation treatment alone. This combined therapeutic effects of IRP2 inhibition and radiation treatment were observed in parental and radioresistant cancer cells, significantly enhancing the proportion of cell death. In conclusion, this study proposes that the genetic or pharmacological inhibition of IRP2 in BC cells may serve as a novel therapeutic strategy for increasing radiosensitivity and overcoming resistance by inducing mitochondrial dysfunction.

## Introduction

Iron is essential for cellular functions vital to growth, and its requirement increases in cancer cells because of its high proliferation and metabolic activity [1, 2]. In addition to facilitating mitochondrial activity and energy production, iron is essential for DNA synthesis, repair, and cell cycle progress [3]. The significance of mitochondria in bioenergetics is well established; however, their role in iron metabolism, which is essential for cellular stress responses and DNA damage repair, has only recently been recognized as a critical factor for tumor cell survival [4–6]. Mitochondrial dysfunction arising from intracellular iron deficiency impairs these pathways and affects cancer cell proliferation and resistance.

Iron Regulatory Protein 2 (IRP2) is a crucial modulator of intracellular iron equilibrium, affecting mitochondrial function and extensive physiological mechanisms that promote cell viability, such as DNA synthesis, repair, and cell cycle regulation [5, 7]. The regulatory role of IRP2 in mitochondrial iron metabolism renders it a pivotal element in the resistance of cancer cells to treatments, such as chemotherapy and radiation, owing to its influence on pathways that might promote tumor growth and recurrence [8–10].

Recent high-throughput screening of an 8-million compound library identified IRP2 inhibitors, indicating that genetic deletion or pharmacological inhibition of IRP2 inhibits the growth of colorectal cancer (CRC) cells, underscoring the therapeutic potential of targeting IRP2 in oncology [11]. This discovery has created new opportunities for cancer treatment, particularly for cancers with dysregulated iron metabolism.

Breast cancer (BC), one of the leading causes of mortality in women under 45 years of age, remains a priority for research aimed at developing targeted personalized therapies to minimize adverse effects and improve survival [12, 13]. Radiation therapy (RT) is essential for managing high-risk BC cases, particularly those with lymph node involvement, triple-negative BC, and HER2-positive subtypes [14, 15]. However, DNA repair capabilities and mutational patterns, lead to varied responses to RT [16–18]. Augmented DNA repair mechanisms correlate with radiation insensitivity and resistance, resulting in suboptimal clinical outcomes in certain patients. This resistance has resulted in an increased interest in combination therapies incorporating immunotherapies or targeted agents to enhance the radiation response [19, 20].

This study examined IRP2 as a potential target to improve the efficacy of RT in BC. Considering the strong correlation between IRP2 and gene sets associated with radiation resistance, altering IRP2 expression may diminish the mechanisms contributing to resistance, thus enhancing cellular sensitivity to radiation. We showed that IRP2 suppression led to mitochondrial dysfunction via iron depletion, leading to cancer cell death. Significantly, IRP2 inhibitors successfully suppressed BC cell proliferation in several models, including BC cell lines, radiation-resistant cell lines, and tumor xenografts, while enhancing sensitivity to radiation. These findings highlight the potential of IRP2 as a target for mitigating radiation resistance in BC and establish a basis for innovative therapeutic options to enhance the efficacy of RT.

## Results

### IRP2 expression is a crucial determinant in modulating radiation sensitivity

Previous studies have shown that disrupting iron metabolism to modulate IRP2 induces mitochondrial dysfunction and that a small-molecule inhibitor targeting IRP2 demonstrates effective tumor-suppressive responses [11]. Given that excessive mitochondrial activation is a poor prognostic factor in therapies such as chemotherapy and RT, we focused on the correlation between IRP2 and key genes involved in antitumor resistance mechanisms.

**Fig. 1:**
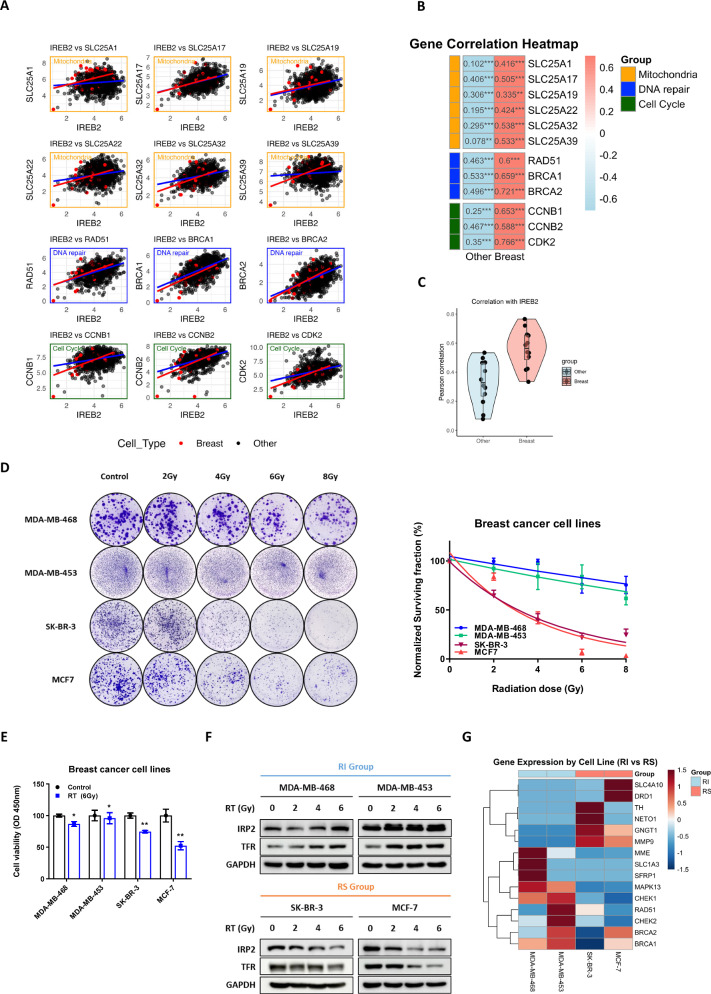
IRP2 directly induces radiation sensitivity in breast cancer (BC) cells. **A** Correlation graph between IREB2 and gene expression related to mitochondria, DNA repair and cell cycle pathways (DepMap Public, 24Q2). **B** Heatmap showing differences in Pearson scores with their p-value between IREB2 and each gene, categorized by lineage (***p < 0.001, **p < 0.01, *p < 0.05) (*n* = 3). **C** Changes in Pearson scores for the mitochondrial gene module following lineage. **D** (Left) Colony formation of BC cells across a dose range of 0 to 8 Gy irradiation, and (Right) the normalized surviving fraction. Data are represented as the mean ± SD (*n* = 3). **E** Cell viability analysis after treatment with radiation for 48 h using the CCK-8 (Cell Counting Kit-8) assay. Data are represented as the mean ± SD. **F** Western blot analysis showing protein levels of IRP2 and TFR in Groups RI and RS treated with radiation for 72 h in a dose-dependent manner. **G** The heatmap represents the differences in gene expression between the RI group and RS group in the response to radiation and DNA repair pathways (*n* = 3).

To explore the role of IRP2 expression in radiation-induced mechanisms, we analyzed the correlations between IREB2 expression and mitochondrial gene modules (SLC25A1, SLC25A22, SLC25A17, SLC25A19, SLC25A32, and SLC25A39), DNA repair genes (RAD51, BRCA1, and BRCA2), and G2M checkpoint genes (CCNB1, CCNB2, and CDK2) using data from the CCLE database on the DepMap portal (Fig. 1A). The Pearson correlation scores for DNA repair genes RAD51, BRCA1, and BRCA2 were notably high, with a marked shift in scores in BC lineages compared to that of the other lineages (Figs. 1B, S1A). The correlation with SLC25A mitochondrial carrier family genes significantly increased, particularly for SLC25A1, which is recognized as a contributor to radiation resistance. Although SLC25A1 typically exhibited no correlation with IREB2 expression across the various lineages, its Pearson score was fourfold higher in the BC lineage (Fig. 1C, S1A). We hypothesized that inhibition of IRP2, which induces mitochondrial dysfunction, would be more effective in BC, prompting an investigation into the relationship between IRP2 and RT in BC cell lines.

To elucidate the relationship between IRP2 expression and the radiation response, we classified four BC cell lines based on their radiation sensitivity. Radiation was delivered at a consistent dose rate across all experiments. Short- and long-term effects were assessed by cell viability assays (Fig. 1E) and clonogenic assays (Fig. 1D), respectively. These analyses demonstrated that MDA-MB-468 and MDA-MB-453 cells were relatively insensitive to radiation doses ranging from 0 to 8 Gy, whereas SK-BR-3 and MCF-7 cells exhibited radiation sensitivity (RS). Based on these data, we categorized MDA-MB-468 and MDA-MB-453 as radiation-insensitive (RI) and SK-BR-3 and MCF-7 as RS for comparative analysis.

We assessed the response of the four BC cell lines to irradiation. In the RI group, the expression of IRP2 and other iron metabolism-related proteins (TFR) increased in a dose-dependent manner. However, the RS group exhibited a negative association between IRP2 expression and radiation dosage (Fig. 1F). We conducted gene pathway analysis to further distinguish between the groups. Gene Set Enrichment Analysis (GSEA) of RNA sequencing data indicated that the “Response to Radiation” pathway was markedly upregulated in the RI group relative to that in the RS group (Fig. S1B). Significantly, DNA repair genes such as CHEK1, CHEK2, BRCA1, and BRCA2 were substantially activated in the RI group (Fig. 1G).

These findings indicate that IRP2 may influence radiation sensitivity in BC cells and highlight its potential involvement in radiation resistance mechanisms via the activation of genes associated with mitochondrial function and the DNA damage response.

### Targeted inhibition of IRP2 enhances radiation sensitivity in BC cells

**Fig. 2:**
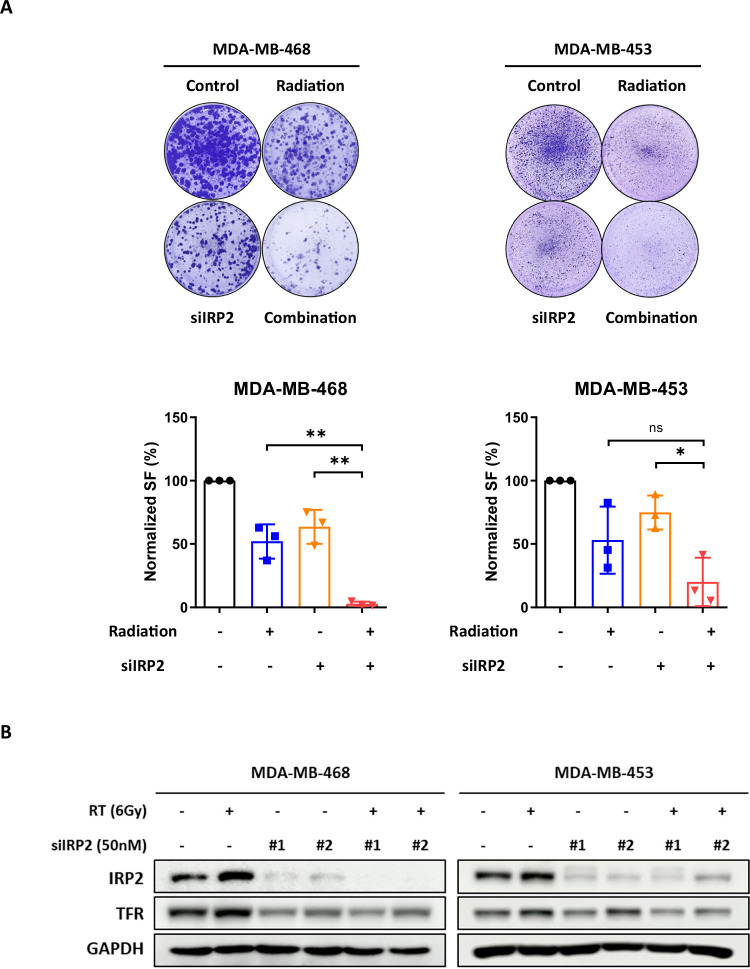
IRP2 silencing enhances radiation sensitivity in BC cells. **A** Colony formation of MDA-MB-468 and MDA-MB-453 (RI group) cells treated with radiation and siIRP2 alone, or in combination with pretreatment of siIRP2 for 48 h. Data are represented as the mean ± SD (*n* = 3). *p < 0.05, **p < 0.01 (*n* = 3). **B** Western blot analysis of MDA-MB-468 and MDA-MB-453 cells after radiation for 48 h, following transfection with 50 nM of siIRP2 for 24 h.

We further investigated whether IRP2 suppression increases radiation sensitivity in BC cells. In the RI group, the administration of siIRP2 prior to RT markedly decreased the normalized surviving fraction in both cell lines. RT alone preserved cell survival by approximately 50%, whereas IRP2 depletion reduced the survival rate to approximately 2% (Fig. 2A). Moreover, siIRP2 pretreatment significantly inhibited the radiation-induced elevation of IRP2 protein levels (Fig. 2B). These data support our hypothesis that IRP2 inhibition enhances radiation sensitivity in BC therapy.

### IRP2 suppression causes mitochondrial dysfunction, DNA Repair, G2/M arrest, autophagy, and BC cell growth inhibition

**Fig. 3:**
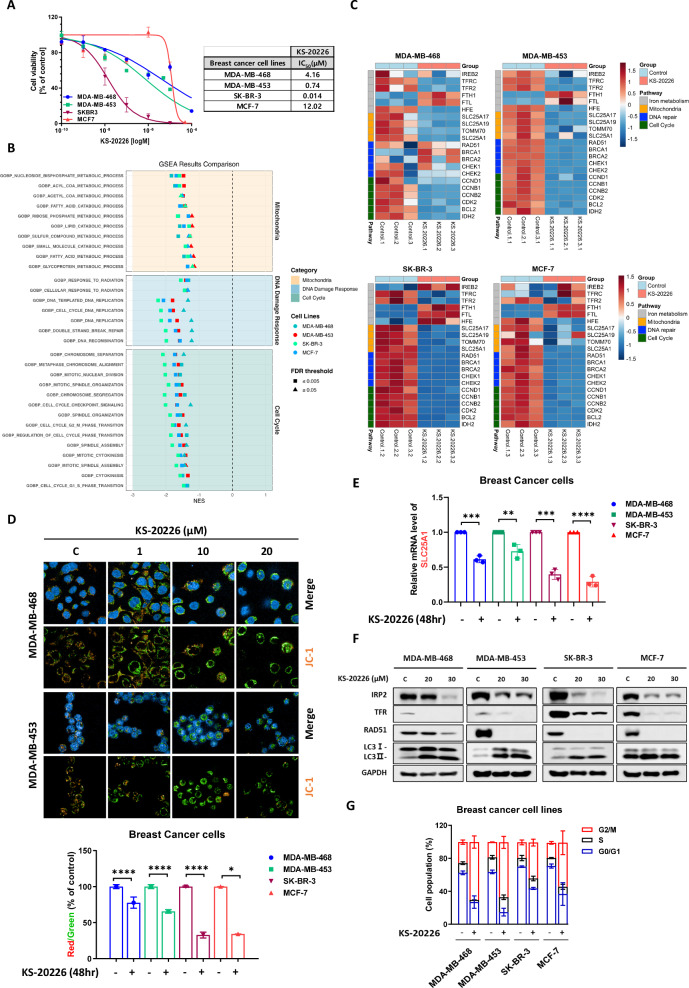
KS-20226 triggers BC cell death via inhibition of IRP2. **A** BC cells are exposed to various concentrations of KS-20226 and then subjected to the CCK-8 assay. The IC50 values for each cell line are presented in the table shown as part of this figure panel. **B** The enrichment NES score plot from GSEA pathway analysis for the GOBP pathway in BC cells (*n* = 3). **C** Heatmap of key genes related to the mitochondrial function, DNA repair, and cell cycle pathways in BC cells (*n* = 3). **D** (Top) JC-1 staining showing mitochondrial dysfunction in RI group treated with KS-20226 in a dose-dependent manner for 48 h. (Bottom) Graph represents Red/Green fluorescence ratio in BC cells treated with 20 µM of KS-20226 (*n* = 3). **E** qRT-PCR results representing the mRNA expression level of SLC25A1 in BC cells after treatment with 20 or 30 µM of KS-20226 for 48 h. Data are represented as the mean ± SD (*n* = 3). **p < 0.01, ***p < 0.001, ****p < 0.001. **F** MDA-MB-468, MDA-MB-453, SK-BR-3, and MCF-7 cells are treated with 20 or 30 µM of KS-20226 for 72 h. Western blotting was performed using lysates with GAPDH as a loading control. **G** Cell cycle analysis of BC cells after treatment with KS-20226 for 48 h, using PI staining and flow cytometry.

Based on previous findings that IRP2-targeting drugs promote autophagy via mitochondrial dysfunction in CRC [11], we investigated whether this finding was also observed in BC cell lines. Initially, we assessed the cytotoxic effects of the IRP2 inhibitor, KS-20226, and found that it markedly diminished the viability of all four breast cancer cell lines (Fig. 3A), with IC_50_ values demonstrating a negative correlation with basal IRP2 protein expression (Fig. S2A). To elucidate the effects of IRP2 inhibition in BC cells, we performed RNA sequencing of MDA-MB-468, MDA-MB-453, SK-BR-3, and MCF-7 cells treated with KS-20226. GSEA indicated that gene sets associated with mitochondrial function, DNA Damage Response (DDR), and cell cycle regulation were markedly downregulated in all the BC cell lines (Figs. 3B, S2B). A heatmap (Fig. 3C) depicts alterations in pivotal pathway genes, such as the SLC25A family genes, RAD51, BRCA1, BRCA2, CCNB1, CCNB2, and CDK2, which are substantially correlated with IREB2 expression in BC cell lines (Fig. S1A).

To complement these transcriptomic findings and verify the functional consequences of mitochondrial dysfunction, we further assessed ROS generation. Using DCFDA and MitoSOX staining, we observed a significant increase in overall ROS and mitochondrial superoxide levels, respectively, across all four parental BC cell lines analyzed (MDA-MB-468, MDA-MB-453, SK-BR-3, and MCF-7). These findings suggest that KS-20226 treatment promotes oxidative stress and mitochondrial dysfunction, as evidenced by elevated ROS levels (Fig. S2C).

Based on these findings, we next sought to directly assess mitochondrial dysfunction. JC-1 staining was used to evaluate the mitochondrial membrane potential, and revealed that with increasing concentrations of KS-20226, green fluorescence (JC-1 monomer) increased, whereas red fluorescence (JC-1 aggregate) decreased, signifying mitochondrial dysfunction (Figs. 3D, S2D). In addition, KS-20226 effectively inhibited mRNA levels of SLC25A1 in both groups of BC cells (Fig. 3E).

Analysis of protein levels following treatment with KS-20226 demonstrated a reduction in IRP2 and TFR levels in all BC cell lines, signifying the successful induction of iron deficiency. Consistent with the RNA sequencing findings, RAD51 expression was also diminished (Fig. 3B, C). The noted elevation in the autophagy marker LC3B indicated that iron-deficiency-induced autophagy was stimulated in BC cells, similar to the findings in CRC (Fig. 3F). Subsequent cell cycle analysis revealed that KS-20226 treatment increased the G2/M phase cell population from 39.8% to 68.1% in the RS group, and from 58.2% to 76.2% in the RI group (Fig. 3G, S2E). This indicates that IRP2 inhibition promotes G2/M arrest more effectively in the RI group than that in the RS group, suggesting a heightened potential for radiation sensitization when targeting the RI group.

These data indicate that IRP2 inhibition enhances the DDR and G2/M arrest via mitochondrial dysfunction, resulting in autophagy and suppression of BC cell proliferation.

### Inhibition of IRP2 increases radiation sensitivity in BC cells via mitochondrial dysfunction

**Fig. 4:**
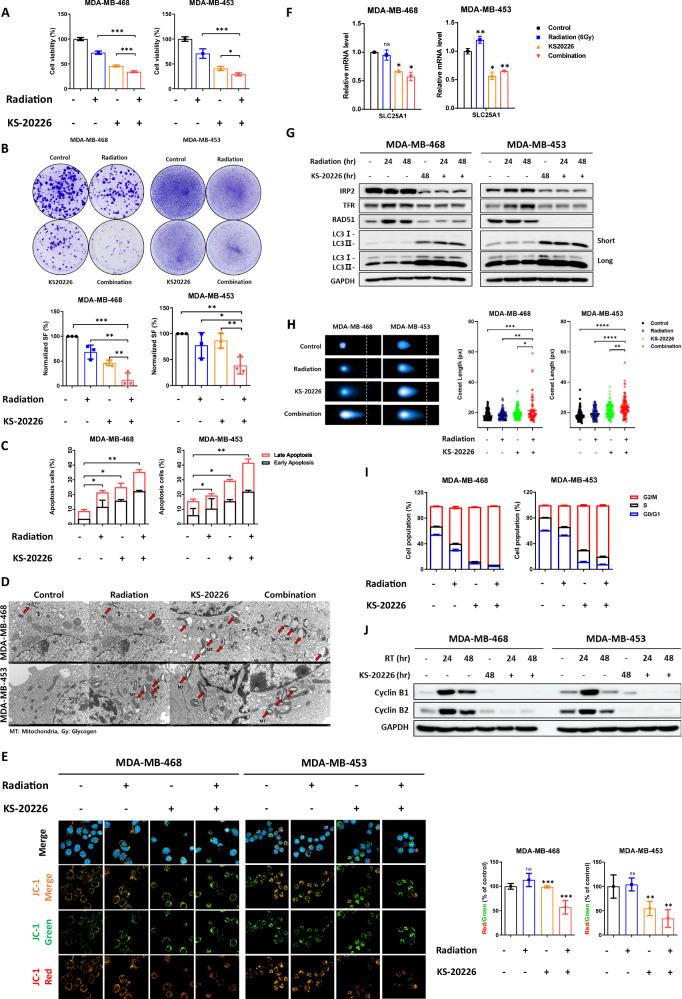
KS-20226 enhances sensitivity to irradiation by inducing apoptosis in BC cells. **A** Cell viability in the RI group of cells (MDA-MB-468, MDA-MB-453) incubated with increasing concentrations of radiation and KS-20226 for 48 h. Data are represented as the mean ± SD (*n* = 3). *p < 0.05, ***p < 0.001. **B** Clonogenic survival assay in MDA-MB-468 and MDA-MB-453 cells treated with radiation alone, KS-20226 alone (0.5 µM), or in combination. Data are represented as the mean ± SD (*n* = 3). *p < 0.05, **p < 0.01, ***p < 0.001. **C** Percentage of apoptosis in MDA-MB-468 and MDA-MB-453 cells treated with radiation, KS-20226, or in combination for 48 or 72 h, followed by flow cytometry analysis. Data are represented as the mean ± SD (*n* = 3). *p < 0.05, **p < 0.01. **D** Representative TEM images of cells treated with 6 Gy radiation and/or 20 μM KS-20226 for 24 h. Red arrows indicate mitochondria (MT) or glycogen (Gy). **E** (Left) Confocal images and (Right) graph showing the JC-1 aggregates-to-monomers ratio in MDA-MB-468 and MDA-MB-453 cells treated with KS-20226 and radiation. Data are represented as the mean ± SD (*n* = 3). **p < 0.01, ***p < 0.001 (*n* = 3). **F** qRT-PCR results representing the mRNA expression level of SLC25A1 in the RI group after treatment with 20 or 30 µM of KS-20226 and 6 Gy of radiation for 72 h. Data are represented as the mean ± SD (*n* = 3). *p < 0.05, **p < 0.01. **G** Protein levels of IRP2, TFR and RAD51 in MDA-MB-468 and MDA-MB-453 cells after treatment with KS-20226 and/or radiation for 48 or 72 h. **H** Comet assay following 24 h treatment with 6 Gy radiation and/or 20 μM KS-20226. (Left) Representative images of comet tails. (Right) Quantification of comet tail length. *p < 0.05, **p < 0.01, ***p < 0.001, ****p < 0.0001. **I** Cell cycle analysis after treatment with KS-20226 and radiation for 48 h, using PI staining and flow cytometry (*n* = 3). **J** Western blots showing changes in protein levels related to the G2/M cell cycle.

To determine whether IRP2 inhibition markedly enhanced radiation sensitivity in BC cells, we initially examined the cytotoxic effects of combined treatment of radiation and KS-20226 in MDA-MB-468 and MDA-MB-453 cells, both of which were categorized in the RI group. This enabled us to confirm the effects of the mitochondrial dysfunction. Initially, the cell viability was approximately 70% after radiation treatment alone; however, following IRP2 depletion, the cell viability decreased to approximately 30% (Fig. 4A). Prolonged treatments significantly reduced the survival proportion (Fig. 4B) and elevated apoptosis by as much as 45% (Fig. 4C).

To investigate whether mitochondrial dysfunction was involved, we next assessed changes in intracellular iron levels. A Calcein-AM fluorescence assay showed that KS-20226 treatment, either alone or in combination with radiation, reduced intracellular labile iron levels, further supporting mitochondrial dysfunction as a downstream event (Fig. S3A). To further substantiate this mechanism, we conducted supplementary experiments evaluating ATP production and the expression of genes related to oxidative phosphorylation. These results showed that KS-20226 significantly reduced mitochondrial ATP production and suppressed the expression of key ETC-related genes, consistent with mitochondrial dysfunction (Fig. S3B, S3C).

Based on these biochemical findings, we further examined the structural integrity of mitochondria through TEM imaging. In the control group, mitochondria maintained a normal structure, whereas radiation alone induced partial cristae disruption, mild swelling, and glycogen accumulation. KS-20226 treatment, either alone or in combination with radiation, markedly exacerbated mitochondrial damage, leading to extensive swelling, rupture, and fragmentation, along with a reduction or complete loss of glycogen. Notably, mitochondrial damage was most severe in the combination group, highlighting the synergistic effect of KS-20226 and radiation in promoting mitochondrial dysfunction (Fig. 4D).

Finally, to directly link mitochondrial dysfunction to the radiation sensitization effect, we used JC-1 staining to demonstrate that KS-20226 decreases mitochondrial membrane potential (MMP) in conjunction with radiation. In the RI group, radiation alone did not reduce MMP; however, the incorporation of KS-20226 markedly increased the green fluorescence intensity in both cell types, signifying augmented mitochondrial failure (Fig. 4E). The red/green ratio underscored the function of KS-20226 as a radiation sensitizer via mitochondrial malfunction (Fig. 4E). Variations in the SLC25A1 mRNA levels further corroborated this finding. In MDA-MB-453 cells, SLC25A1 levels exhibited a modest increase following radiation treatment; however, KS-20226 inhibited this increase (Fig. 4F). This confirms the role of SLC25A1 in radiation resistance and demonstrates that IRP2 inhibition improves radiation sensitivity by mitigating the factors associated with radiation resistance via mitochondrial dysfunction.

Subsequent investigation of protein expression demonstrated that IRP2 inhibitors diminished radiation-induced RAD51 levels, impairing DNA repair by inducing iron depletion during DNA damage. This disruption hindered adequate recovery and resulted in autophagic cell death (Fig. 4G). To functionally confirm that impaired DNA repair led to increased DNA damage, we performed comet assays in both MDA-MB-468 and MDA-MB-453 cells.

KS-20226 treatment alone significantly increased comet tail length compared to the control, and this effect was further enhanced in the combination group. The most pronounced increase in comet length was observed in cells treated with both radiation and KS-20226, particularly in the radiation-insensitive cell lines (Fig. 4H). Consistent with these results, increased γH2AX protein levels were also observed by western blot analysis (Fig. S3D). These findings provide strong evidence that KS-20226 effectively promotes DNA damage, especially when combined with radiation. Furthermore, mRNA analysis of the combination therapy indicated that KS-20226 suppressed radiation-induced elevations in IRP2 and TFR levels, while enhancing ATM and ATR expression (Fig. S3E, S3F). Cell cycle studies at the G2/M checkpoint and protein expression verified that IRP2 inhibition triggered G2/M arrest. In the RI group, the percentage of cells in the G2/M phase was maintained at approximately 50% with radiation alone, but considerably increased to 92% in MDA-MB-468 cells upon the addition of KS-20226. A comparable increase was observed in MDA-MB-453 cells (Fig. 4I). Radiation alone increased cyclin B1 and cyclin B2 levels; however, the IRP2 inhibitor impeded this action (Fig. 4J). These data indicate that in RI cells, the G2/M checkpoint protein cyclin B was activated due to radiation resistance; however, the suppression of IRP2 curtailed this activation, extending G2/M phase arrest, and ultimately resulting in cell death. The combination of KS-20226 and radiation decreased the mRNA levels of CCNB1, CCNB2, and CDK1, suggesting disruption of cell cycle checkpoints induced by radiation (Fig. S3G).

These findings indicate that IRP2 inhibition increases radiation sensitivity in RI BC cell lines, suggesting that mitochondrial dysfunction caused by intracellular iron depletion impedes DNA repair, exacerbates DNA damage, and induces cell cycle arrest, ultimately culminating in cell death.

### Combination of IRP2 inhibition and radiation significantly inhibits tumor growth in MDA-MB-468 cells

**Fig. 5:**
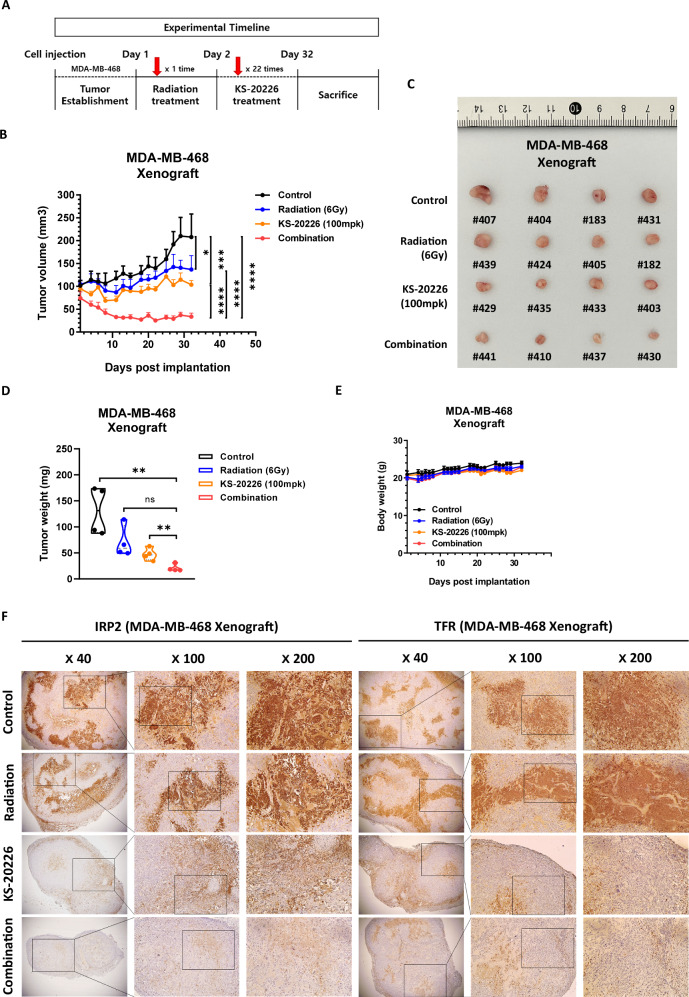
Combination therapy of KS-20226 and radiation effectively inhibits tumor growth in MDA-MB-468. **A** Schematic illustration of the experimental timeline for in vivo treatment. **B** Tumor volumes in MDA-MB-468 xenograft mouse models treated with vehicle, radiation (6 Gy), KS-20226 (100 mg/kg), and combination treatment (6 Gy + KS-20226 100 mg/kg) for 32 days. Data are represented as the mean ± SD (*n* = 4). *p < 0.05, ***p < 0.001, ****p < 0.001. **C** Tumor images from the MDA-MB-468 xenograft model. **D** Tumor weights after treatment for 32 days with KS-20226 in MDA-MB-468 xenografts. Data are represented as the mean ± SD (*n* = 4). **p < 0.01. **E** Mouse body weight after treatment for 32 days with KS-20226 in MDA-MB-468 xenografts. **F** Representative IHC images of tumor tissues stained for indicated targets. Images were acquired at 40×, 100×, and 200× magnifications.

After identifying KS-20226 as a radiation sensitizer in BC cell lines, we assessed its antitumor activity in an in vivo xenograft model of BC cells. In the MDA-MB-468 xenograft model, therapy commenced with X-Rad at a dose of 6 Gy concurrently with KS-20226 at 100 mg/kg on the same day. KS-20226 was injected five times weekly for a total of 22 doses (Fig. 5A). Observations over 32 days indicated that the combination therapy of KS-20226 and radiation markedly diminished tumor growth relative to each treatment administered independently, with tumors in the combination cohort exhibiting no growth (Fig. 5B). Moreover, both the tumor size (Fig. 5C) and weight (Fig. 5D) were significantly reduced in the combination treatment group relative to those in the individual treatment groups. Despite the significant synergistic effect of radiation and the IRP2 inhibitor on tumor growth inhibition, no overt toxicity was observed in any treatment group, as assessed by body weight and general appearance throughout the study period (Figs. 5E, S4A). As an in vivo validation of our proposed mechanism, we performed immunohistochemical analysis of IRP2 and TFR in tumor tissues from MDA-MB-468 xenografts. KS-20226 treatment markedly reduced the expression levels of both IRP2 and TFR (Figs. 5F, S4B). Notably, TFR levels were further decreased in the combination group, suggesting that KS-20226 enhances tumor sensitivity to radiation (Fig. S4B). These findings support the proposed mechanism of radiosensitization via IRP2 inhibition and iron depletion.

### Inhibition of IRP2 promotes apoptosis in radiation-resistant (RR) BC cells

**Fig. 6:**
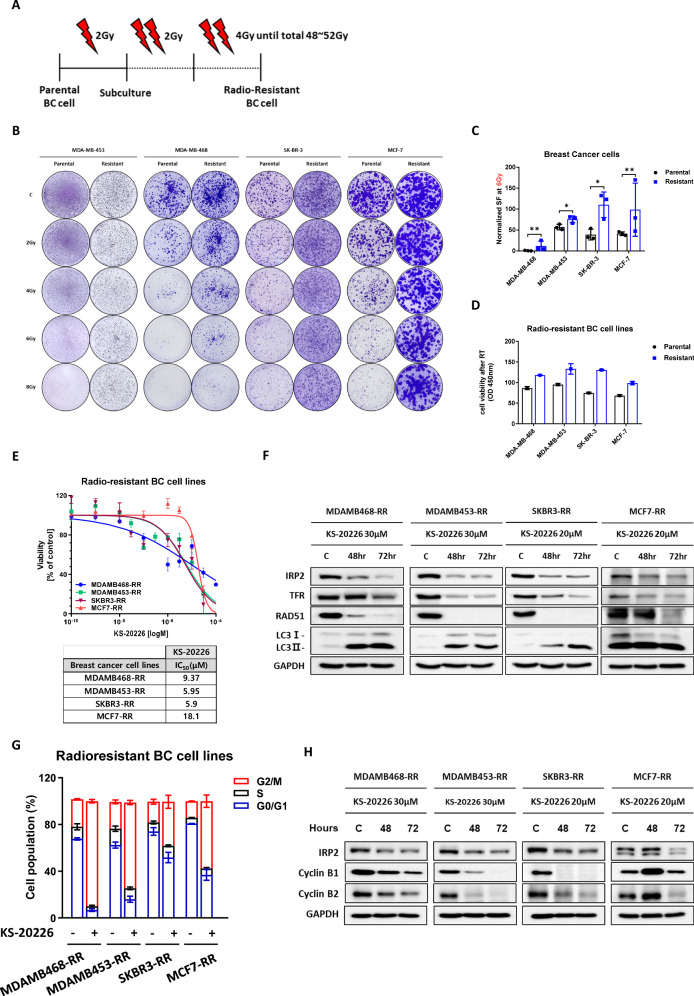
KS-20226 significantly inhibits the cell viability of radiation-resistant cells. **A** Methodology for establishing radiation-resistant cell lines. **B** Colony formation of parental and radiation-resistant BC cells across a dose range of 0 to 8 Gy irradiation. **C** Normalized surviving fraction of parental and resistant BC cells after 6 Gy of irradiation. Data are represented as the mean ± SD (*n* = 3). *p < 0.05, **p < 0.01. **D** CCK-8 assay results for parental and radiation-resistant breast cancer cells after 6 Gy irradiation for 48 h, compared to parental cells. **E** Cell viability of radioresistant BC cells treated with KS-20226, as summarized in the table included within the figure. **F** Protein levels in radioresistant BC cells after time dependent treatment with KS-20226. **G** Changes in G2/M cell population in resistant cells after treatment with KS-20226. **H** Western blots showing changes in protein levels related to the G2/M cell cycle after treatment with KS-20226 for 48 h.

Considering that IRP2 inhibition influences the factors associated with chemotherapeutic resistance, we investigated the possibility of using IRP2 inhibitors as radiation sensitizers and treatments to mitigate radiation resistance. To ascertain the efficacy of KS-20226 in targeting RR BC cells, we initially developed RR cells by incrementally exposing the parental BC cells to radiation. We initiated treatment with a low dose of 2 Gy, which was subsequently increased to 4 Gy after reaching cumulative doses of 48 or 52 Gy, resulting in the development of four RR BC cell lines from the parental lines (Fig. 6A).

To verify the establishment of resistance, we performed colony formation tests (Fig. 6B, C) and evaluated the viability of both parental and RR cells following radiation treatment in a dose-dependent manner (Figs. 6D, S5A). Following the assessment of resistance, we analyzed the radiation response in the parental and RR cells within the RS group (SK-BR-3 and MCF-7). Consistent with our expectations, IRP2 and TFR levels exhibited bidirectional modulation in response to radiation. In parental cells subjected to 4 Gy of radiation, IRP2 and TFR were downregulated in a time-dependent manner, corroborating with previous findings (Fig. 1G). The expression of these proteins was markedly elevated in RR cells (Fig. S5B), reflecting the increase observed in the RI group (MDA-MB-468 and MDA-MB-453) following radiation exposure (Fig. 1G). These findings highlight the significance of IRP2 in the development of radiation resistance.

Analysis of cell viability in RR BC cells demonstrated that KS-20226 significantly reduced cell viability, with IC_50_ values substantiating its effectiveness even in resistant cells (Fig. 6E). Consistent with the observations in parental BC cell lines, treatment with KS-20226 also induced a significant increase in intracellular ROS and mitochondrial superoxide levels in the four reprogrammed resistant (RR) cell lines, as confirmed by DCFDA and MitoSOX staining (Fig. S5C). Additionally, KS-20226 also effectively downregulated mRNA levels of SLC25A1 in RR BC cells (Fig. S5D). Protein analysis indicated that KS-20226 markedly downregulated IRP2, TFR, and RAD51, while upregulating LC3B (Fig. 6F), in accordance with the responses noted in parental cells (Fig. 5). Furthermore, KS-20226 induced G2/M arrest in RR cells by suppressing the production of cyclins B1 and B2 (Fig. 6G, H), which validated our previous findings (Fig. 5).

In summary, IRP2 inhibitors induce iron deficiency in cancer cells, resulting in mitochondrial dysfunction and cell mortality by impairing DNA repair and disrupting cell cycle checkpoints, thereby enhancing radiation sensitivity.

Importantly, these effects were consistently observed not only in parental radiation-insensitive (RI) cells but also in reprogrammed resistant (RR) cells, supporting the therapeutic potential of IRP2 inhibition as a strategy to overcome resistance during radiotherapy in breast cancer.

## Discussion

Targeting iron metabolism offers a promising approach for cancer therapy; however, nonselective iron chelators are constrained by potential side effects and toxicity resulting from their interactions with other metal ions. Water-soluble iron chelators such as deferoxamine and deferasirox exhibit in vitro efficacy but are hindered by low membrane permeability and the formation of reactive oxygen species [21, 22]. Our research demonstrates that IRP2 inhibitors specifically target IRP2, obstructing its interaction with IRE, leading to cellular apoptosis and inhibition of tumor proliferation while reducing systemic iron metabolism. Given that IRP2 expression corresponds to the efficacy of various inhibitors, IRP2 may function as a predictive biomarker, as evidenced by our findings (Fig. S2A). Previous studies have shown that IRP2 specifically modulates iron metabolism in CRC and has prognostic relevance [11]. IRP2 inhibitors exhibit antitumor properties in patient-derived cell lines and in vivo models, diminishing oxidative phosphorylation (OXPHOS) and promoting autophagy through the AMPK-ULK1-Beclin1 pathway [14, 23].

Mitochondrial iron metabolism is significantly enhanced in cancer cells, aiding in resistance to anticancer therapy; thus, inducing mitochondrial dysfunction through IRP2 inhibition is a prospective area of research [8, 24]. Our gene correlation analysis between IRP2 and therapy resistance-related genes revealed a robust association with genes implicated in homologous recombination repair, cell cycle regulation, and mitochondrial citrate carrier proteins, with notably elevated correlation scores in BC cell lines. In addition to direct effects on DNA repair pathways, it is important to consider the role of iron-sulfur (Fe-S) cluster-containing enzymes in maintaining genomic stability. Iron depletion induced by KS-20226 could disrupt the function of Fe-S cluster-dependent DNA repair proteins, thereby further compromising genome integrity and exacerbating DNA damage [25, 26]. SLC25A1, a mitochondrial citrate transporter within the SLC25A family, is pivotal for autophagy, redox homeostasis, and mitochondrial metabolism, influencing BC tumorigenesis and radiation resistance [27–29].

In line with this, our ultrastructural analysis revealed that radiation-induced mitochondrial stress was accompanied by glycogen accumulation, a feature commonly associated with radiation resistance (Fig. 4D). KS-20226 treatment, particularly in combination with radiation, markedly disrupted mitochondrial integrity and abolished glycogen accumulation. These observations suggest that KS-20226 may counteract radiation-induced metabolic adaptation, further contributing to the reversal of radiation resistance [30, 31].

This study combined the capacity of IRP2 inhibitors to induce mitochondrial dysfunction and diminish OXPHOS with strategies aimed at improving the efficacy of RT. In vitro research on radiation responsiveness in BC cell lines revealed a correlation between IRP2 expression and radiation sensitivity, indicating that modulation of IRP2 could transform RR cells into more RS cells. Consistent with this concept, genetic suppression of IRP2 enhanced radiation sensitivity in the RI group, essentially resensitizing resistant cells. RNA sequencing analysis demonstrated that the pharmacological suppression of IRP2 by KS-20226 resulted in the downregulation of mitochondrial gene modules, DNA repair, and G2/M checkpoint gene sets in various BC cell lines, a conclusion corroborated by assessments of mitochondrial function and the cell cycle. KS-20226 significantly suppressed cell proliferation in vitro, demonstrating its effectiveness as a radiation sensitizer in combination therapy in the RI group. KS-20226 effectively triggers apoptosis in BC cells and induces complete radiation resistance. Collectively, our findings indicate that IRP2 represents a significant target for radiation sensitization and that specific IRP2 inhibitors, in contrast to conventional iron chelators, may surmount resistance to RT.

Despite these encouraging results, our study has some limitations that necessitate additional investigation. A comprehensive analysis of radiation-responsive genes is required. While we investigated the impact of IRP2 inhibition using RNA sequencing in CRC and BC cells, we did not have genomic data for cancer cells exclusively subjected to radiation [32, 33]. Administering consistent radiation doses to both the RI and RS groups, in conjunction with the IRP2 inhibition data, may elucidate the function of IRP2 in augmenting radiation sensitivity.

However, additional studies are required to improve the efficacy and safety evaluation of these inhibitors. Although KS-20226 exhibited no observable toxicity in animal studies—based on body weight and physical appearance—comprehensive histopathological analysis of major organs such as the liver and kidney was not conducted, representing a limitation of the current study. Future investigations should include detailed organ toxicity assessments and long-term safety profiling to better characterize the therapeutic window of KS-20226. Moreover, while the compound demonstrates potent in vitro activity, its pharmacological efficacy in vivo is relatively modest [34, 35]. Therefore, optimizing its chemical properties, such as improving solubility, may enhance its antitumor efficacy at lower doses and reduce the risk of off-target effects in future preclinical models.

One limitation of this study is the use of a single high-dose (6 Gy) irradiation in the in vivo experiments, rather than a clinically relevant fractionated regimen (e.g., 2 Gy × 3). This choice was based on in vitro observations that 6 Gy, rather than 2 Gy, was necessary to induce a robust increase in IRP2 and TFR expression in MDA-MB-468 cells. Nonetheless, future studies incorporating fractionated irradiation protocols may further validate and extend our findings under more clinically applicable conditions.

Our findings highlight the potential of IRP2 inhibition to augment radiation sensitivity in cancer cells, particularly to overcome radiation resistance in BC cells. Future investigations should examine the synergistic effects of IRP2 inhibitors in conjunction with RT through a comprehensive analysis, thereby facilitating their clinical use. This study enhances our understanding of the biological importance of IRP2 in cancer therapy and establishes a basis for novel therapeutic approaches aimed at addressing radiation resistance mechanisms with the objective of creating more effective cancer treatments.

## Methods and materials

### Cell culture

MDA-MB-453, SK-BR-3, and MCF-7 human BC cells were purchased from the Korean Cell Line Bank (Seoul, South Korea). MDA-MB-468 cells were purchased from (American Type Culture Collection ATCC (Manassas, VA, USA)). MDA-MB-453, SK-BR-3, and MCF-7 cells were cultured in RPMI (Roswell Park Memorial Institute) 1640 medium (Lonza, Basel, Switzerland) supplemented with 10% fetal bovine serum, 100 U/mL penicillin, and 100 µg/mL streptomycin at 37 °C in a 5% CO_2_ atmosphere. MDA-MB-468 cells were cultured in DMEM (Dulbecco’s Modified Eagle Medium) medium (HyClone Laboratories Inc, Utah, USA) supplemented with 10% fetal bovine serum, 100 U/mL penicillin, and 100 µg/mL streptomycin at 37 °C and with 5% CO_2_.

### Cell viability assay

Cell viability was assessed using a Cell Counting Kit-8 (CCK-8; Dojindo, Kumamoto, Japan). The cells were seeded in 96-well culture plates 1.5 × 10^4^ cells/well. After incubation to allow cell adhesion to the bottom of the plate, KS-20226 and radiation were applied to individual wells, followed by incubation at 37 °C. After 48 h, CCK-8 reagent (10 μL/well) was added per well and incubated for 3–4 h at 37 °C. The absorbance was measured using an ELISA reader (VERSA Max, Molecular Devices, LLC. 3860 N First Street San Jose, CA 95134) at a wavelength of 450 nm. The IC_50_ values were calculated using GraphPad Prism software (San Diego, CA, USA).

### Western blot

Protein expression was measured using western blotting. The cells were harvested by scraping and lysed using radioimmunoprecipitation assay buffer (ELPIS-BIOTECH, Daejeon, South Korea) containing phosphatase and protease inhibitors (GenDEPOT). The following primary antibodies were used: IRP-2 (7H6) (SC-33682, Santa Cruz, Dallas, Texas 75220 USA), CD71 (SC-32272, Santa Cruz), RAD51 (8875S, Cell Signaling, Danvers, Massachusetts, USA), LC3B (2775S, Cell Signaling), Cyclin B1 (SC-245, Santa Cruz), Cyclin B2 (SC-28303, Santa Cruz).

### Flow cytometry

To analyze the apoptotic cells (%), 0.8 × 10^6^ cells were seeded in 60 mm cell culture dish and incubated at 37 °C. After 24 h, the cells were treated with KS-20226 and radiation, alone or in combination. Cells were then collected by trypsinization and centrifugation. Cells were resuspended in 100 μL of 1X binding buffer and stained with the phycoerythrin (PE) Annexin V Apoptosis Detection Kit (Thermo Scientific, USA), according to the protocol provided by the manufacturer. They were then gently vortexed and incubated for 15 min at room temperature (RT, 21–25 °C) in the dark. After incubation, 3–400 μL of 1X binding buffer was added. For cell cycle analysis, cells were washed with PBS and stained with propidium iodide (PI) and RNase A staining buffer (Becton, Dickinson and Company, New Jersey, USA) for 15 min at RT in the dark. The stained cells were analyzed using a BD FACSymphony A5 (Becton Dickinson company). Flow software (Turku Bioscience, Turku, Finland) was used for data analysis.

### Clonogenic assay

For the clonogenic assay, we seeded 5 × 10^3^–2 × 10^4^ BC cells in 35 mm or 60 mm culture dish and incubated them for 2–3 weeks until colonies formed in the absence or presence of treatment. The medium was changed every 2–3 days after the colonies stabilized on the plates, and only colonies with > 40 cells were counted. After colony formation, the cells were washed gently with 1X PBS, fixed, and stained using a crystal violet mixture containing 4% paraformaldehyde and 100% methanol for 30 min at RT. The number of colonies was analyzed using the ImageJ software (National Institutes of Health, Bethesda, MD, USA). To normalize BC cells with different plating efficiencies, the plating efficiency (PE) was calculated to estimate the surviving fraction (SF). The formula used for the analysis is as follows:

$${\rm{Surviving\; fraction}}({\rm{SF}})={\rm{number\; of\; colonies\; formed\; after\; treatment}}/{\rm{number\; of\; cells\; seeded}}\times 100 \% {\rm{;plating\; efficiency}}({\rm{PE}})={\rm{number\; of\; colonies\; formed\; in\; untreated\; cells}}/{\rm{number\; of\; cells\; seeded}}\times 100 \%$$

### Transfection

Cells were seeded and cultured at a density of 0.8 × 10^6^ cells/well in 60 mm culture plates. After the cells reached 60–70% confluency, they were transfected with targeted siRNA using Lipofectamine RNAiMAX (Invitrogen, USA) according to the protocol provided by the manufacturer for 24 h. Cells were treated with KS20226 or irradiated after transfection. The siRNA was synthesized by Bioneer (Daejeon, South Korea).

RNA Extraction: Cells were seeded and cultured in 60 mm culture plates (0.8 × 10^6^ cells). The cells were treated with KS-20226 and radiation alone or in combination. After 48–72 h, total RNA was extracted using the Ribospin2 kit (Geneall, Seoul, South Korea) according to the instructions provided by the manufacturer. The RNA quality and concentration were measured using a NanoDrop 1000 spectrophotometer (Thermo Scientific, Waltham, MA, USA).

### Quantitative real-time reverse transcription polymerase chain reaction (qRT-PCR)

A total of 1000 ng of extracted RNA in the previous step was reverse transcribed to cDNA using a High-Capacity cDNA Reverse Transcription Kit (Applied Biosystems Inc., MA, USA) according to the protocol provided by the manufacturer. qPCR was performed on a Quantstudio3 (Applied Biosystems) using SYBR Green Master Mix (Applied Biosystems Inc.) following the instructions provided by the manufacturer. β-actin was used as the endogenous control for normalization. The following primers were used for qRT-PCR: IRP1 (Forward primer: 5′-CGCTGTGGTTGACTTTGCTGCAAT-3′, Reverse primer: 5′-ATCTATTACAAGATCAGCAGGGCAG-3′), IRP2 (Forward primer: 5′-GGCTGCAGAGCTGTACCAGAAAGAA-3′, Reverse primer: 5′-CGGTCCTTTGGCAGCCCAGTCTCT-3′), TFR (Forward primer: 5′-ACTTGCCCAGATGTTCTCAG-3′, Reverse primer: 5′-GTATCCCTCTAGCCATTCAGTG-3′), RAD51 (Forward primer: 5′-TTTGGAGAATTCGAACTGG-3′, Reverse primer: 5′-TACATGGCCTTTCCTTCAC-3′), BRCA1 (Forward primer: 5′-GCTCGTGGAAGATTTCGGTGT-3′, Reverse primer: 5′-TCATCAATCACGGACGTATCATC-3′), BRCA2 (Forward primer: 5′-CAGAAGCCCTTTGAGAGTGG-3′, Reverse primer: 5′-TCCATCTGGGCTCCATTTAG-3′), ATM (Forward primer: 5′-GGTATAGAAAAGCACCAGTCCAGTATTG-3′, Reverse primer: 5′-CGTGAACACCGGACAAGAGTTT-3′), ATR (Forward primer: 5′-AGTAGCTTCCTTTCGCTCCAA A-3′, Reverse primer: 5′-ACTGACTCCGGCCACTCCAT-3′), CDK1 (Forward primer: 5′-TGGATCTGAAGAAATACTTGGATTCTA-3′, Reverse primer: 5′-CAATCCCCTGTAGGATTTGG-3′), CCNB1 (Forward primer: 5′-ACAGGTCTTCTTCTGCAGGG-3′, Reverse primer: 5′-GAACTTGAGCCAGAACCTGA-3′), CCNB2 (Forward primer: 5′-ATTTTTACAGGTTCAGCCAG-3′, Reverse primer 5′-ATCTCCTCATACTTGGAAGC-3′), SLC25A1 (Forward primer: 5′-CCCCATGGAGACCATCAAG-3′, Reverse primer: 5′-CCTGGTACGTCCCCTTCAG-3′), SLC25A17 (Forward primer: 5′-GGTGGTAAACACCAGACTGAAGC-3′, Reverse primer: 5′-AGCCGAGATTCCTTCATCGCGA-3′), SLC25A19 (Forward primer: 5′-GCCATACCAGCCGAAGGAAAGA-3′, Reverse primer: 5′-CTCCAACCTGTAGCCGCTTCTT-3′), SLC25A22 (Forward primer: 5′-GTCAACGAGGACACCTACTCTG-3′, Reverse primer: 5′-GGAAGTAGACCACCTGTGCGAT-3′), SLC25A32 (Forward primer: 5′-GCGTCTTATCCAACCTTGCGCT-3′, Reverse primer: 5′-GTTTCCAAATGGTAGTCAAGCAATG-3′), SLC25A39 (Forward primer: 5′-CACTGCCTATGACCAACTGAAGG-3′, Reverse primer: 5′-CTTTGTCCGCATAAGCTCCAGG-3′), and β-actin (Forward primer:5′-TTGCCGACAGGATGCAGAAG-3′, Reverse primer: 5′-AGGTGGACAGCGAGGCCAGG-3′).

### Differential gene expression analysis by RNA sequencing

Public data on gene correlation scores were obtained from the DepMap portal (DepMap Public 24Q2) and subsequently analyzed using the Data Explorer tool and visualized as a scatter plot using R studio (4.1.2). Pathway enrichment was analyzed with GSEA software (v_4.2.3), and “GOBP” was applied to gene set database.

RNA sequencing was performed using total RNA samples from BC cells treated with KS-20226 for 48 h. The basal gene levels and changes in mRNA expression upon treatment with KS-20226 were analyzed using differentially expressed gene and gene enrichment plots were generated using GSEA software.

### Comet assay

MDA-MB-468 and MDA-MB-453 breast cancer cells were treated with 6 Gy of ionizing radiation and 20 μM KS-20226, followed by a neutral Comet assay to evaluate DNA damage. Microscope slides were pre-coated with 1% low-gelling agarose. Treated cells were harvested using trypsinization, washed with cold PBS, and adjusted to a concentration of 4 × 10⁴ cells/mL. A total of 400 μL of the cell suspension was mixed with 1.2 mL of 1% low-gelling agarose and layered onto the pre-coated slides. Slides were then incubated in neutral lysis buffer overnight at 4 °C in the dark. After lysis, the slides were rinsed three times with TBE buffer for 30 min at room temperature and subjected to electrophoresis in TBE buffer for 20 min. The slides were fixed in 70% ethanol for 30 min at room temperature, air-dried in the dark, and stained with DAPI (Sigma, St. Louis, MO, USA). Imaging was performed using an Operetta CLS high-content imaging system (PerkinElmer, USA), and DNA damage was quantified using CometScore 2.0 software.

### Live cell imaging assay for ROS and mitochondrial membrane potential

Live-cell imaging was used to assess intracellular ROS levels and mitochondrial membrane potential in breast cancer cells. For ROS analysis, eight breast cancer cell lines (four parental and four radiation-resistant) were treated with 20 μM KS-20226. After 24 h, cells were stained with 10 μM H2DCFDA (Invitrogen™, D399) or 5 μM MitoSOX™ Red (Invitrogen™, M36008) in serum-free medium for 30 min at 37 °C in the dark.

For mitochondrial membrane potential assessment, cells were seeded in 96-well plates and stained with a pre-warmed solution containing 0.2 mg/mL JC-1 and 0.1 mg/mL Hoechst 33342. Cells were incubated at 37 °C for 20–30 min in a humidified incubator with 5% CO₂, washed with PBS, and imaged immediately.

All images were acquired using an Operetta CLS high-content imaging system (PerkinElmer, USA), and fluorescence intensities (including red/green JC-1 ratio) were quantified using Harmony software.

### Irradiation

For in vitro experiments, cells were irradiated using a Gammacell Low Dose-Rate Research Irradiator (MDS Nordion, Canada) at a dose rate of approximately 3.2 Gy/min. Cells were exposed to single doses of 2, 4, or 6 Gy, and subsequent experiments, including cell viability assays and clonogenic assays, were conducted to assess radiation responses at short- and long-term timepoints, respectively.

For in vivo experiments, mice bearing MDA-MB-468 xenografts were irradiated with a single dose of 6 Gy using an X-Rad irradiator (Precision, North Branford, CT, USA) under identical dose rate conditions.

### Transmission electron microscopy (TEM)

Transmission Electron microscopy Specimens were fixed for 12 h in 2% Glutaraldehyde-2% Paraformaldehyde in 0.1 M phosphate buffer(pH 7.4) and washed in 0.1 M phosphate buffer, post-fixed with 1% OsO4 in 0.1 M phosphate buffer for 2 hr and dehydrated with an ascending ethanol series(50, 60, 70, 80, 90, 95, 100, 100%) for 10 min each, and infiltrated with propylene oxide for 10 min. Specimens were embedded with a Poly/Bed 812 kit, polymerized in an electron microscope oven at 70 °C for 12 hr. The block is equipped with a diamond knife in the Ultra-microtome(UC7) and is cut into 200 nm semithin section and stained toluidine blue for observation of light microscope. The region of interest was then cut into 80 nm thin sections using the ultra-microtome, placed on copper grids, double stained with 5% Uranyl acetate for 20 min and 3% Lead citrate for 7 min staining, and imaged with a transmission electron microscopy(HT7800) at the acceleration voltage of 100 kV equipped with a RC camera.

### ATP production assay

The ATP production rate was measured using the Seahorse XF Real-Time ATP Rate Assay (Agilent Technologies). MDA-MB-468 and MDA-MB-453 cells were seeded at a density of 4 × 10⁴ cells per well in XF24 cell culture plates and treated with 6 Gy radiation followed by 20 μM KS-20226 for 24 h. Cells were incubated in Seahorse XF base medium supplemented with 10 mM glucose and 2 mM glutamine. The assay was conducted using sequential injections of oligomycin (1 μM) and rotenone/antimycin A (0.5 μM), according to the manufacturer’s instructions. ATP production through mitochondrial respiration and glycolysis was calculated using Wave software. Normalization was performed using Hoechst 33342 nuclear staining and cell count.

### Animal models and radiation exposure

To investigate the effect of KS-20226 and radiation on tumor growth in vivo, MDA-MB-468 cell suspensions mixed with Matrigel at a 1:1 ratio (1.5 × 10^7^ cells/mL) were injected into 6-week-old female nude mice (Central Lab. Animal, Inc., Seoul, South Korea). The number of animals per group was determined based on prior studies and was considered sufficient to detect meaningful differences in tumor growth. Mice with tumor volumes of approximately 80–100 mm^3^ were randomly assigned to the experimental and vehicle control groups. Mice that did not develop measurable tumors (~100 mm³) prior to treatment were excluded based on a pre-established criterion. The mice were then treated with 6 Gy radiation (X-Rad, Precision, North Branford, CT, USA), KS-20226 (100 mg/kg; 5 times/week), or a combination of both. Tumor volume and body weight were assessed three and five times per week, respectively. Tumor volume was calculated as follows: Tumor volume = length (mm) × width^2^ (mm^2^) × 1⁄2. The animal study was conducted in accordance with Institutional Animal Care and Use Committee requirements (2021-0102).

For in vitro experiments, cells were treated with 2, 4, or 6 Gy of radiation using a Gammacell Low Dose-Rate Research Irradiator (MDS Nordion, Canada).

### Immunohistochemistry (IHC)

Formalin-fixed, paraffin-embedded tumor tissues were sectioned (4 μm), deparaffinized, and rehydrated through graded ethanol. Antigen retrieval was performed using 10x citrate buffer (pH 6.0; Sigma, C9999) in an autoclave. Endogenous peroxidase activity was blocked with 3% hydrogen peroxide in methanol. After blocking with normal horse serum in PBS, slides were incubated overnight at 4 °C with primary antibodies against IRP2 and transferrin receptor (TFR) (1:500 dilution). Detection was performed using a DAB Substrate Kit Peroxidase (HRP), with Nickel (Vector Laboratories, SK-4100), followed by hematoxylin counterstaining. Slides were mounted with Epredia™ Mounting Medium (Thermo, 4112), and images were acquired using an Olympus microscope and analyzed using ImageJ.

### Statistical analysis

Statistical analyses were performed using GraphPad Prism (v5.01) software for each experiment. The data included at least three replicates for each experiment and are presented as mean ± S.D. Statistical comparisons were analyzed using an unpaired two-tail t-test and one-way ANOVA, with significance levels indicated in the graphs as follows: *p < 0.05, **p < 0.01, ***p < 0.001 and ****p < 0.0001. Sample sizes were determined to ensure sufficient power to detect statistically significant differences based on prior experiments and effect size expectations.

## Supplementary information

Supplementary data

Original western blots
