# Supplementary material for: Targeting iron regulatory protein 2 (IRP2) to disrupt iron metabolism enhances radiosensitivity through mitochondrial dysfunction in breast cancer cells
Source: Cell Death Discov. 2025 Jul 31;11:357. doi: 10.1038/s41420-025-02653-z (PMC12314050; doi:10.1038/s41420-025-02653-z)
Supplement: Supplementary file 1 — Supplementary data [file 41420_2025_2653_MOESM1_ESM.docx]

Targeting Iron Regulatory Protein 2 (IRP2) to Disrupt Iron Metabolism Enhances Radiosensitivity Through Mitochondrial Dysfunction in Breast Cancer Cells

Ye Yeong Jeong^1,2^, Jieon Hwang^2^, Areum Park^3,4^, Sungmin Cho^1,2^, Inyoung Cho^2,5^, Soseul Won^2,5^ You Me Shin^1,2^, Sung Eun Kim^1,2^, Chan Hoon Maeng ^1,2^, Jaemoon Yang^6,7^, Minhee Ku^6,7^, Hyuk Lee^3^* and Sang Joon Shin^2,8^*

Supporting Information

Supplement figure

| **A** |  |  |  |  |
| --- | --- | --- | --- | --- |
|  | **Correlation score with IREB2 log2 expression** | | | |
|  | **Lineage** | | | |
|  | **Other** | | **Breast** | |
| **Gene** | **Pearson** | **p-value** | **Pearson** | **p-value** |
| **RAD51** | 0.463 | 5.85E-78 | 0.6 | 8.22E-08 |
| **BRCA1** | 0.533 | 4.81E-107 | 0.659 | 1.35E-09 |
| **BRCA2** | 0.496 | 4.49E-91 | 0.721 | 5.97E-12 |
| **CCNB1** | 0.25 | 4.34E-22 | 0.653 | 2.10E-09 |
| **CCNB2** | 0.467 | 2.72E-79 | 0.588 | 1.64E-07 |
| **CDK2** | 0.35 | 5.90E-43 | 0.766 | 4.06E-14 |
| **SLC25A1** | 0.102 | 1.06E-04 | 0.416 | 4.62E-04 |
| **SLC25A17** | 0.406 | 8.64E-59 | 0.505 | 1.30E-05 |
| **SLC25A19** | 0.306 | 7.91E-33 | 0.335 | 5.54E-03 |
| **SLC25A22** | 0.195 | 7.15E-14 | 0.424 | 3.50E-04 |
| **SLC25A32** | 0.295 | 1.51E-30 | 0.538 | 2.71E-06 |
| **SLC25A39** | 0.078 | 2.83E-03 | 0.533 | 3.39E-06 |


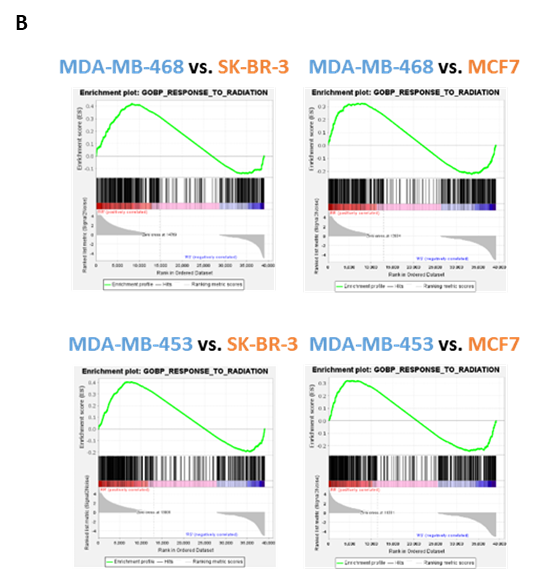


**Supplement Figure 1. Correlation between IREB2 and radiation resistance genes.** (A) Correlation between the gene expression of IREB2 and SLC25A family members, HR repair genes (RAD51, BRCA1, and BRCA2), and G2/M checkpoint proteins (CCNB1, CCNB2, and CDK1) in cancer. A table displaying the Pearson correlation coefficients and p-values for each graph. (B) Enrichment plot comparing the RI and RS groups in response to radiation.

**
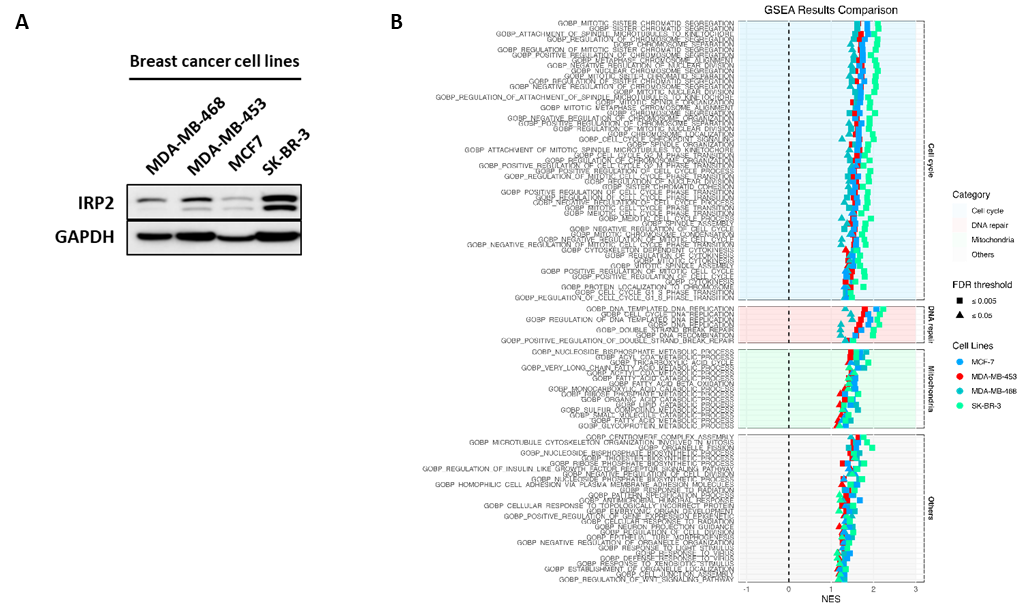
**

**
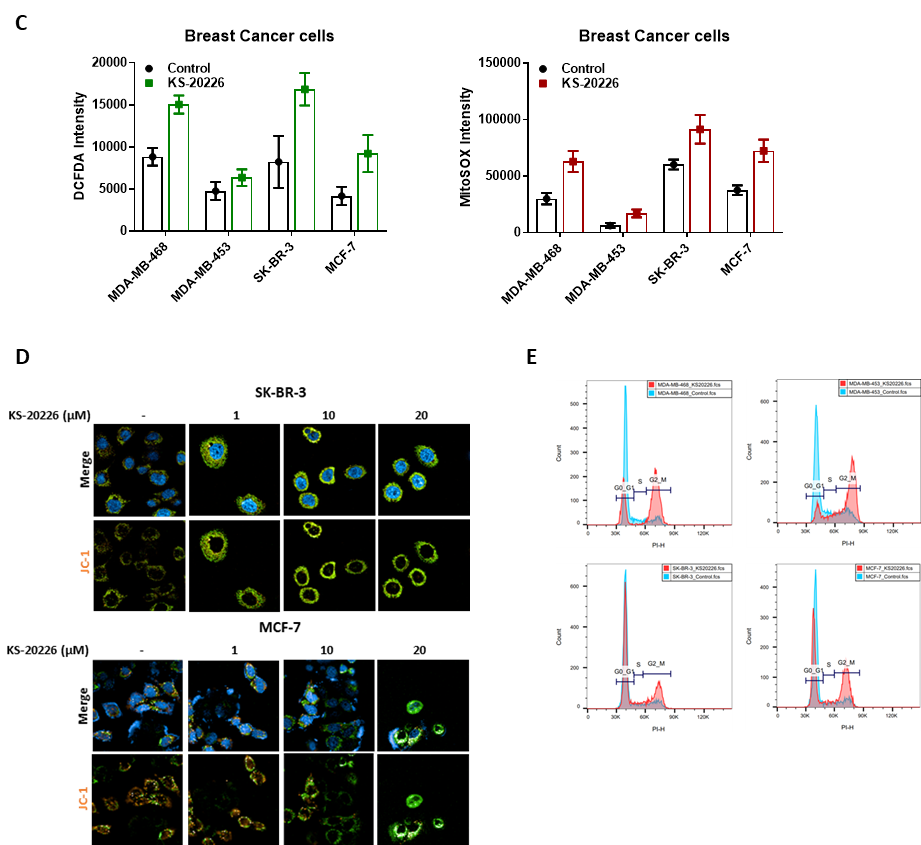
**

**Supplement Figure 2. KS-20226 effectively reduces pathways inducing radioresistance in BC Cells.** (A) Protein expression of IRP2 in BC cells was measured by western blotting. (B) Enrichment NES score plot from GSEA of the GOBP pathway in BC cells. ***p < 0.001, **p < 0.01, *p < 0.05 (n=3). (C) Quantification of DCFDA and MitoSOX fluorescence intensity in breast cancer cell lines after 24 h treatment with KS-20226. (D) JC-1 staining shows mitochondrial dysfunction in SK-BR-3 and MCF-7 cells treated with KS-20226 in a dose-dependent manner (n=3). (E) Cell cycle analysis of BC cells after treatment with KS-20226 for 48 h using PI staining and flow cytometry (n=3).


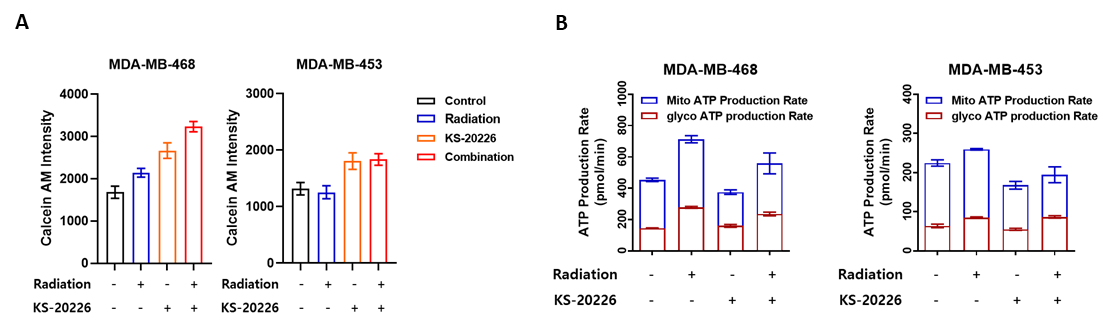


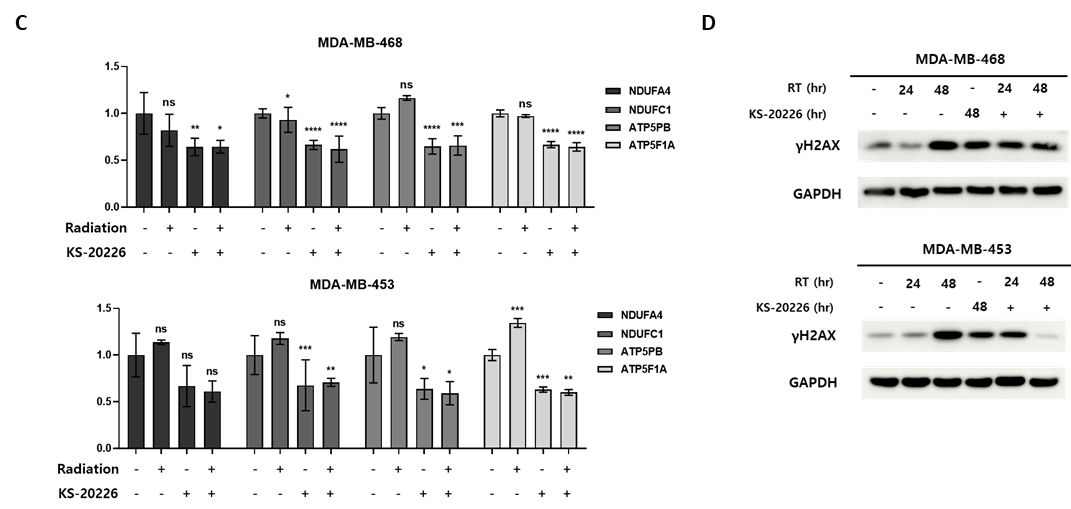


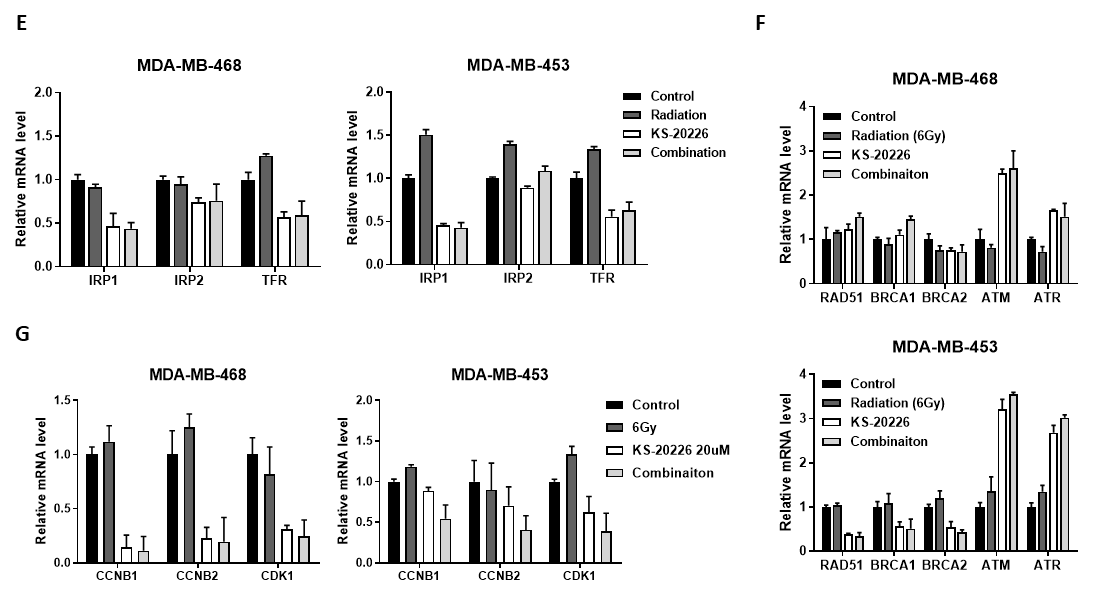


**Supplement Figure 3. Iron depletion occurs with radiation-induced DNA damage and G2/M arrest through mitochondrial dysfunction.** (A) Labile iron pool (LIP) analysis using Calcein AM fluorescence after 24 h combination treatment (6 Gy radiation + 20 μM KS-20226). (B) Mitochondrial ATP production rate measured by ATP assay. (C) qRT-PCR analysis of genes related to mitochondrial ETC and ATP synthesis. (D) Western blot analysis of γH2AX protein levels. (E–G) qRT-PCR analysis of genes related to iron metabolism (E), DNA repair and damage markers (F), and G2/M checkpoint regulators (G) following combination treatment in MDA-MB-468 and MDA-MB-453 cells.

**
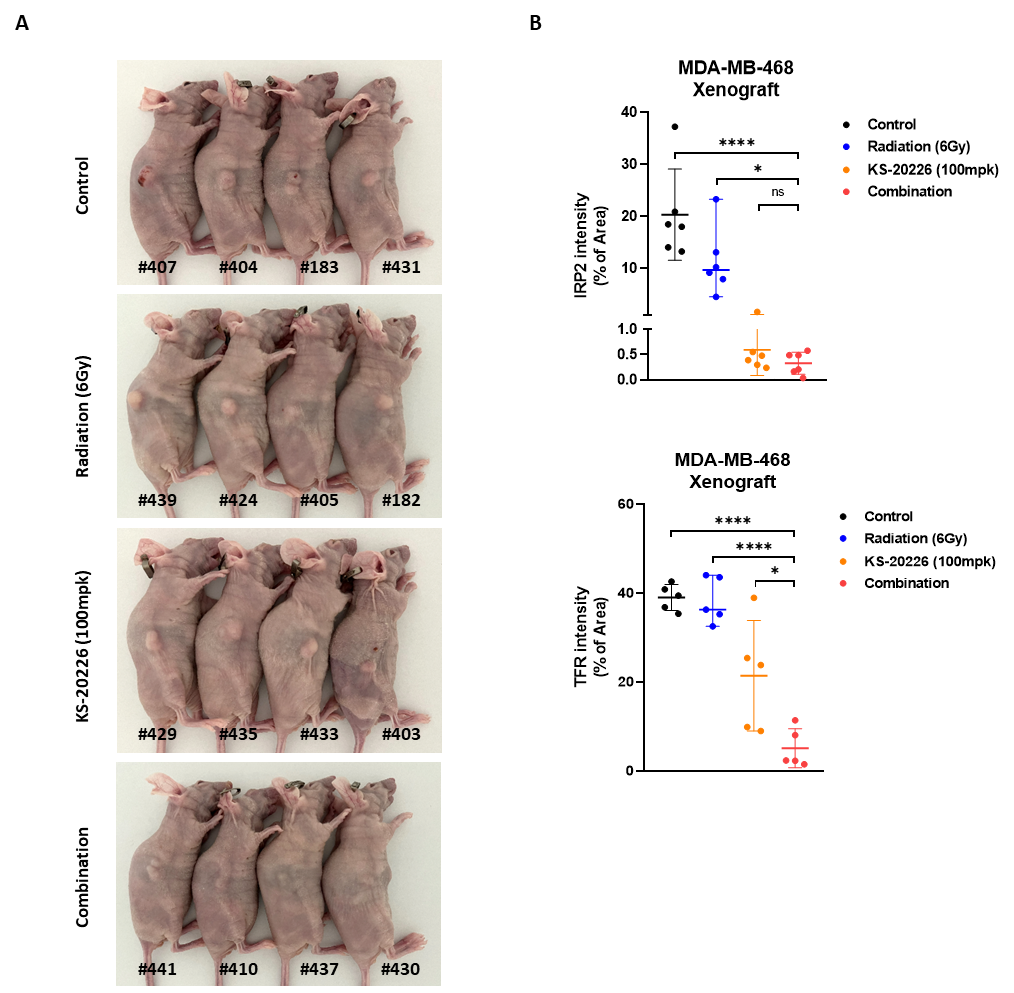
**

**Supplement Figure 4. IRP2 inhibitor sensitizes radiation response *in vivo.*** (A) Images show mice treated with KS-20226 and X-ray irradiation for 32 days (n=4). (B) Quantification of IRP2 and TFR IHC staining intensity. *p < 0.05, ****p < 0.0001.


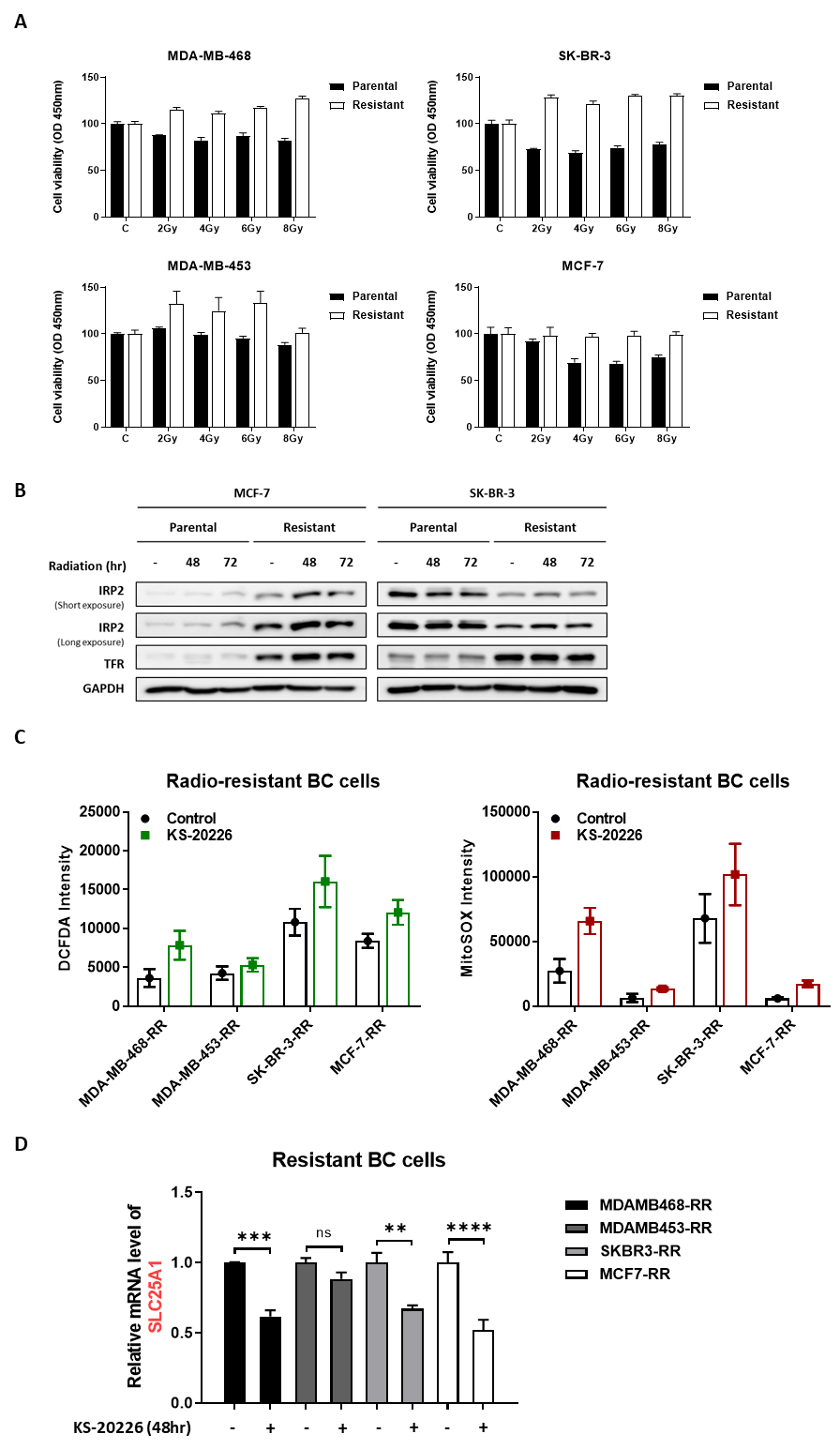


**Supplement Figure 5. Iron metabolism has a strong correlation with radiation resistance.** (A) Cell viability analysis in radiation-resistant BC cells after treatment with radiation for 48 h using the CCK-8 assay. (B) Comparison of iron metabolism-related protein responses between parental and radiation-resistant cell lines after radiation treatment in the RS group. (C) Quantification of DCFDA and MitoSOX fluorescence intensity in radiation-resistant breast cancer cell lines after 24 h treatment with KS-20226 to assess ROS levels. (D) qRT-PCR results representing the mRNA expression level of SLC25A1 in radioresistant BC cells after treatment with 20 or 30 µM of KS-20226 for 48 h. Data are represented as the mean ± SD (n = 3). ***p < 0.001, ****p < 0.0001.
