# Supplementary material for: Targeting iron regulatory protein 2 (IRP2) to disrupt iron metabolism enhances radiosensitivity through mitochondrial dysfunction in breast cancer cells
Source: Cell Death Discov. 2025 Jul 31;11:357. doi: 10.1038/s41420-025-02653-z (PMC12314050; doi:10.1038/s41420-025-02653-z)
Supplement: Supplementary file 2 — Original western blots [file 41420_2025_2653_MOESM2_ESM.pptx]

## Slide 1
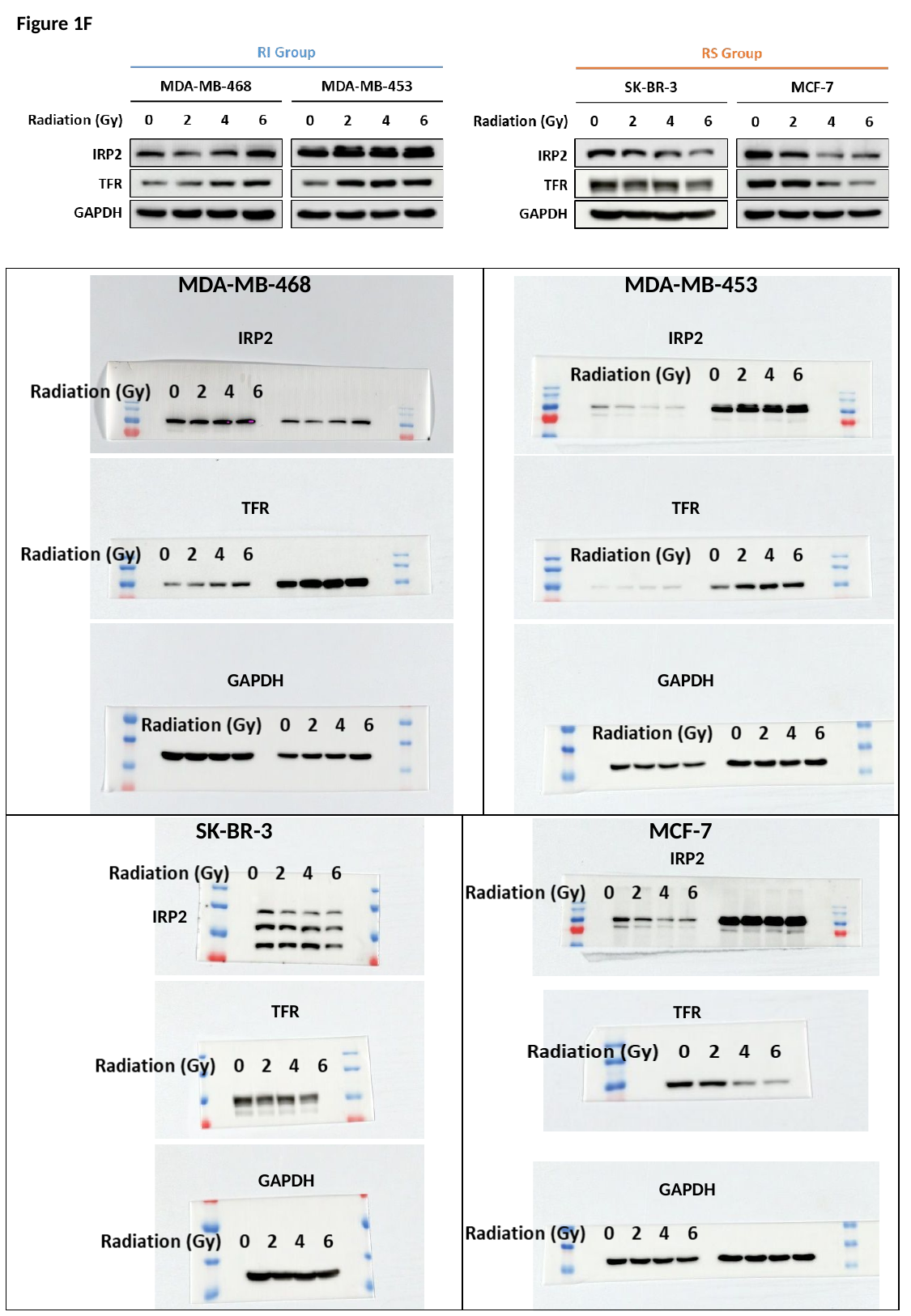

Figure 1F
| MDA-MB-468 |
| --- |
| MDA-MB-453 |
| --- |
| IRP2 |
| --- |
| IRP2 |
| --- |
| TFR |
| --- |
| TFR |
| --- |
| GAPDH |
| --- |
| GAPDH |
| --- |
| MCF-7 |
| --- |
| SK-BR-3 |
| --- |
| IRP2 |
| --- |
| IRP2 |
| --- |
| TFR |
| --- |
| TFR |
| --- |
| GAPDH |
| --- |
| GAPDH |
| --- |

## Slide 2
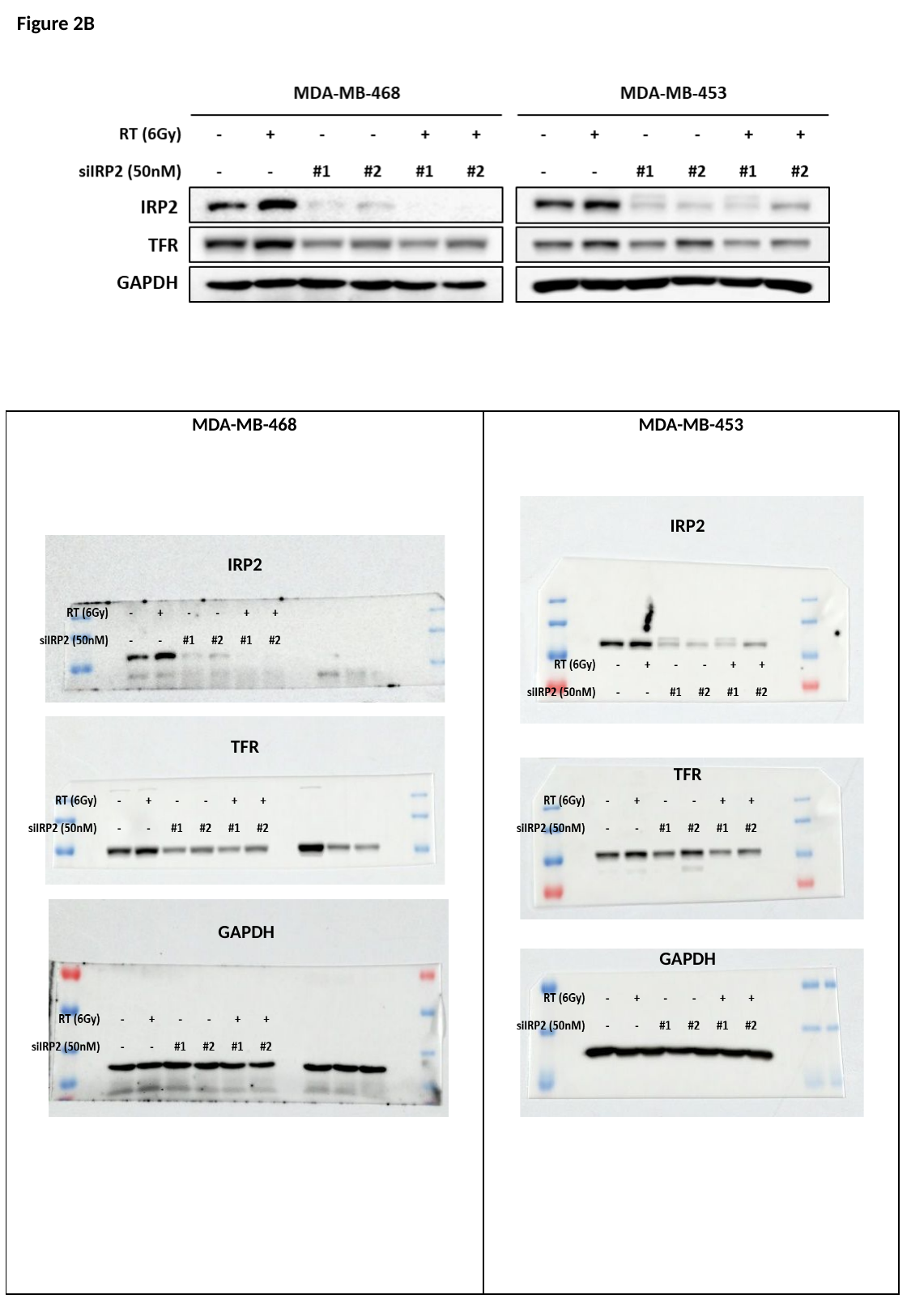

Figure 2B
| MDA-MB-468 |
| --- |
| MDA-MB-453 |
| --- |
| IRP2 |
| --- |
| IRP2 |
| --- |
| TFR |
| --- |
| TFR |
| --- |
| GAPDH |
| --- |
| GAPDH |
| --- |

## Slide 3
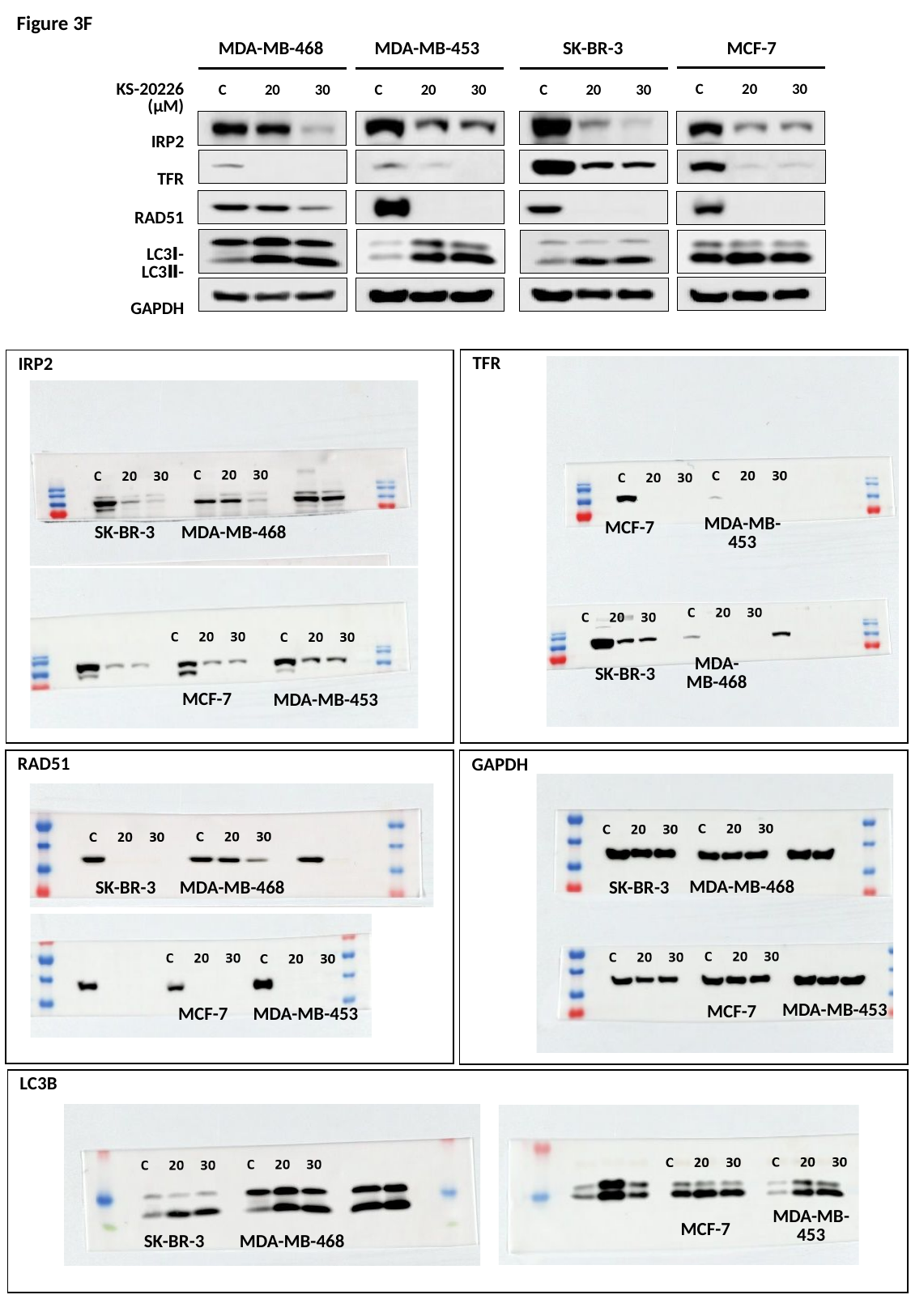

Figure 3F
SK-BR-3
MCF-7
MDA-MB-468
MDA-MB-453
| C | 20 | 30 |
| --- | --- | --- |
| KS-20226 (μM) |
| --- |
| IRP2 |
| TFR |
| RAD51 |
| LC3Ⅰ- LC3Ⅱ- |
| GAPDH |
| C | 20 | 30 |
| --- | --- | --- |
| C | 20 | 30 |
| --- | --- | --- |
| C | 20 | 30 |
| --- | --- | --- |
| TFR |
| --- |
| IRP2 |
| --- |
| MCF-7 |
| --- |
| MDA-MB-468 |
| --- |
| SK-BR-3 |
| --- |
| MDA-MB-453 |
| --- |
| MDA-MB-468 |
| --- |
| SK-BR-3 |
| --- |
| MCF-7 |
| --- |
| MDA-MB-453 |
| --- |
| RAD51 |
| --- |
| GAPDH |
| --- |
| MDA-MB-468 |
| --- |
| SK-BR-3 |
| --- |
| SK-BR-3 |
| --- |
| MDA-MB-468 |
| --- |
| MDA-MB-453 |
| --- |
| MCF-7 |
| --- |
| MDA-MB-453 |
| --- |
| MCF-7 |
| --- |
| LC3B |
| --- |
| MDA-MB-453 |
| --- |
| MCF-7 |
| --- |
| MDA-MB-468 |
| --- |
| SK-BR-3 |
| --- |

## Slide 4
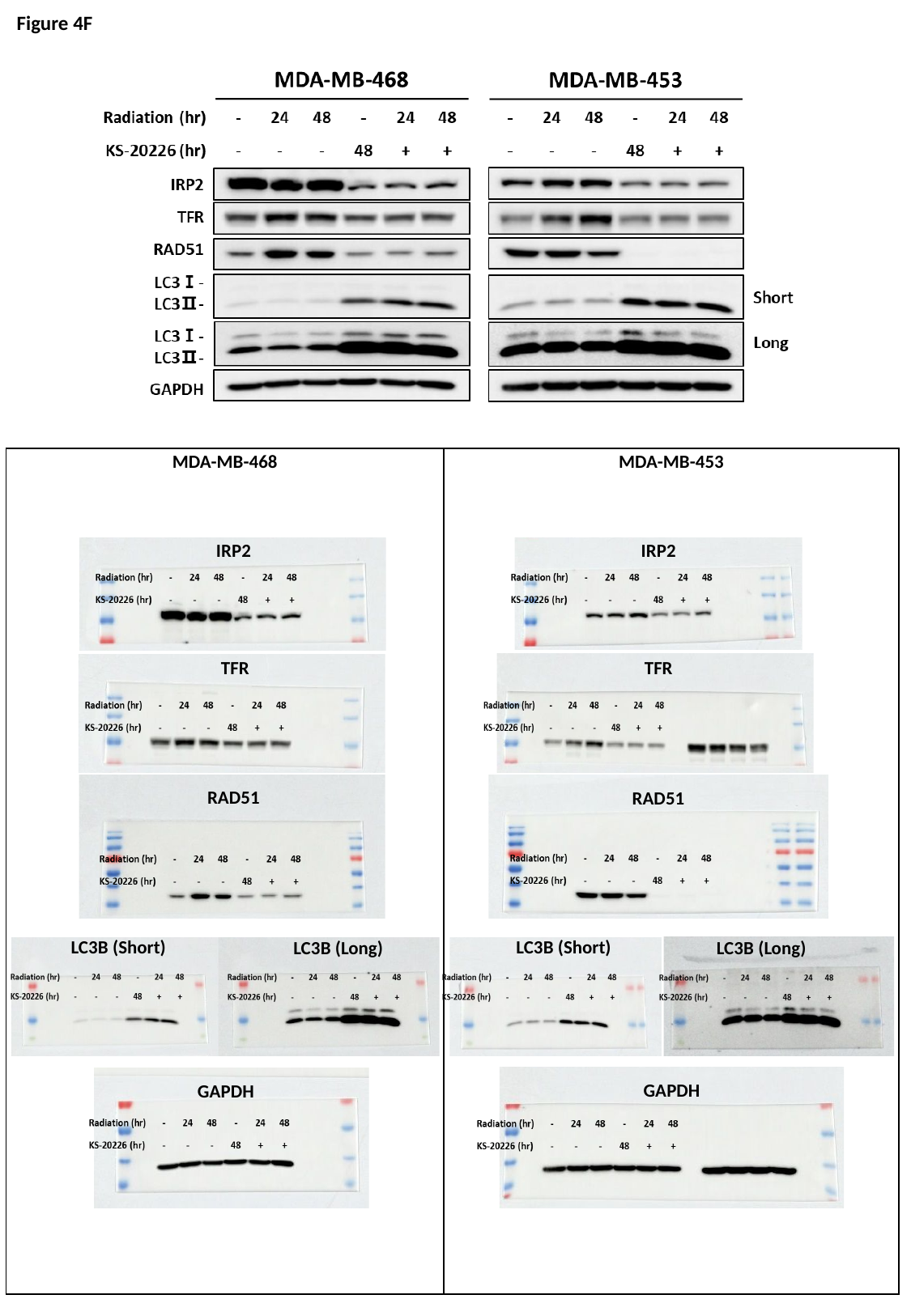

Figure 4F
| MDA-MB-468 |
| --- |
| MDA-MB-453 |
| --- |
| IRP2 |
| --- |
| IRP2 |
| --- |
| TFR |
| --- |
| TFR |
| --- |
| RAD51 |
| --- |
| RAD51 |
| --- |
| LC3B (Short) |
| --- |
| LC3B (Short) |
| --- |
| LC3B (Long) |
| --- |
| LC3B (Long) |
| --- |
| GAPDH |
| --- |
| GAPDH |
| --- |

## Slide 5
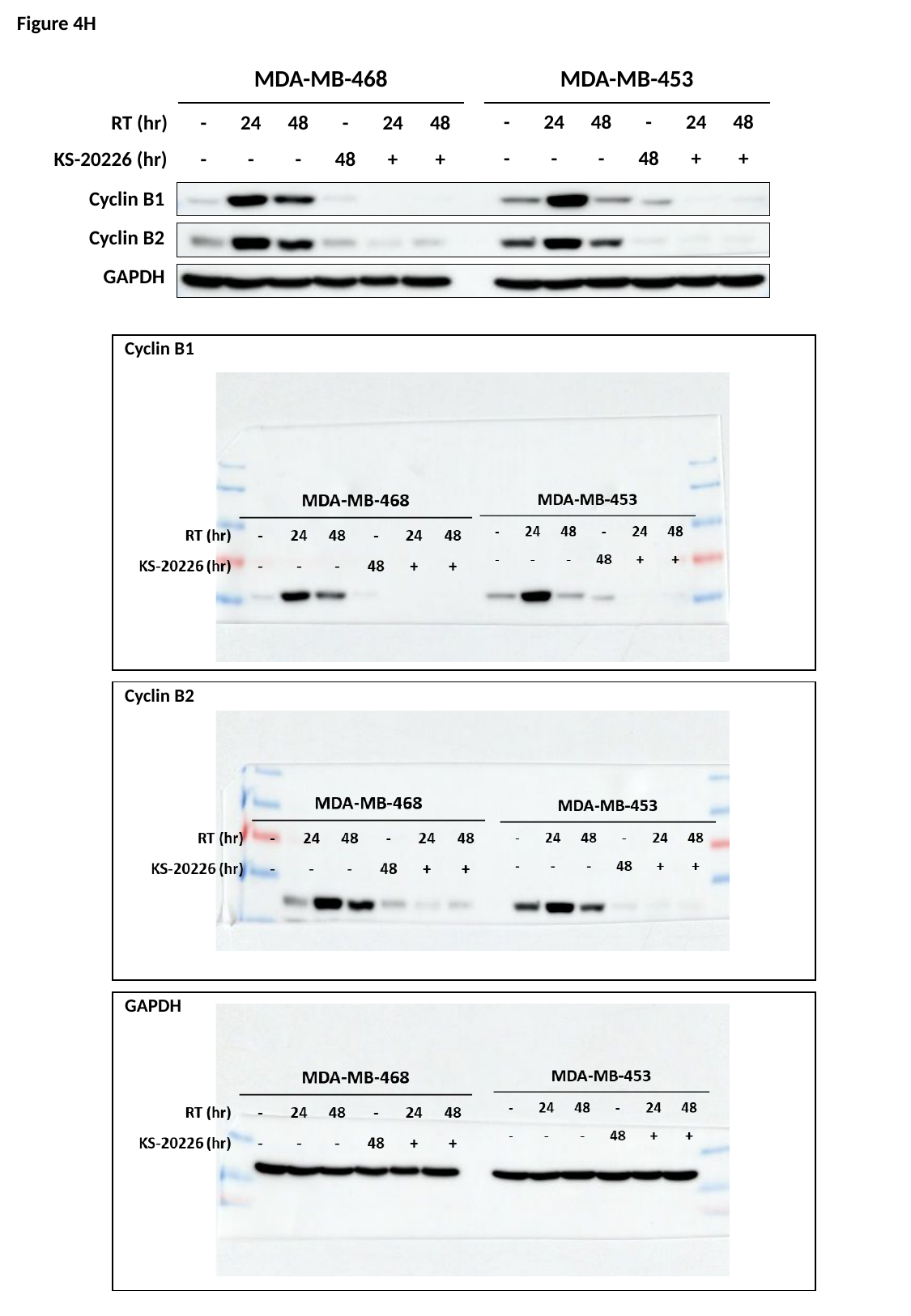

Figure 4H
| MDA-MB-468 |
| --- |
| MDA-MB-453 |
| --- |
| - | 24 | 48 | - | 24 | 48 |
| --- | --- | --- | --- | --- | --- |
| - | - | - | 48 | + | + |
| RT (hr) | - | 24 | 48 | - | 24 | 48 |
| --- | --- | --- | --- | --- | --- | --- |
| KS-20226 (hr) | - | - | - | 48 | + | + |
| Cyclin B1 |
| --- |
| Cyclin B2 |
| GAPDH |
| Cyclin B1 |
| --- |
| Cyclin B2 |
| --- |
| GAPDH |
| --- |

## Slide 6
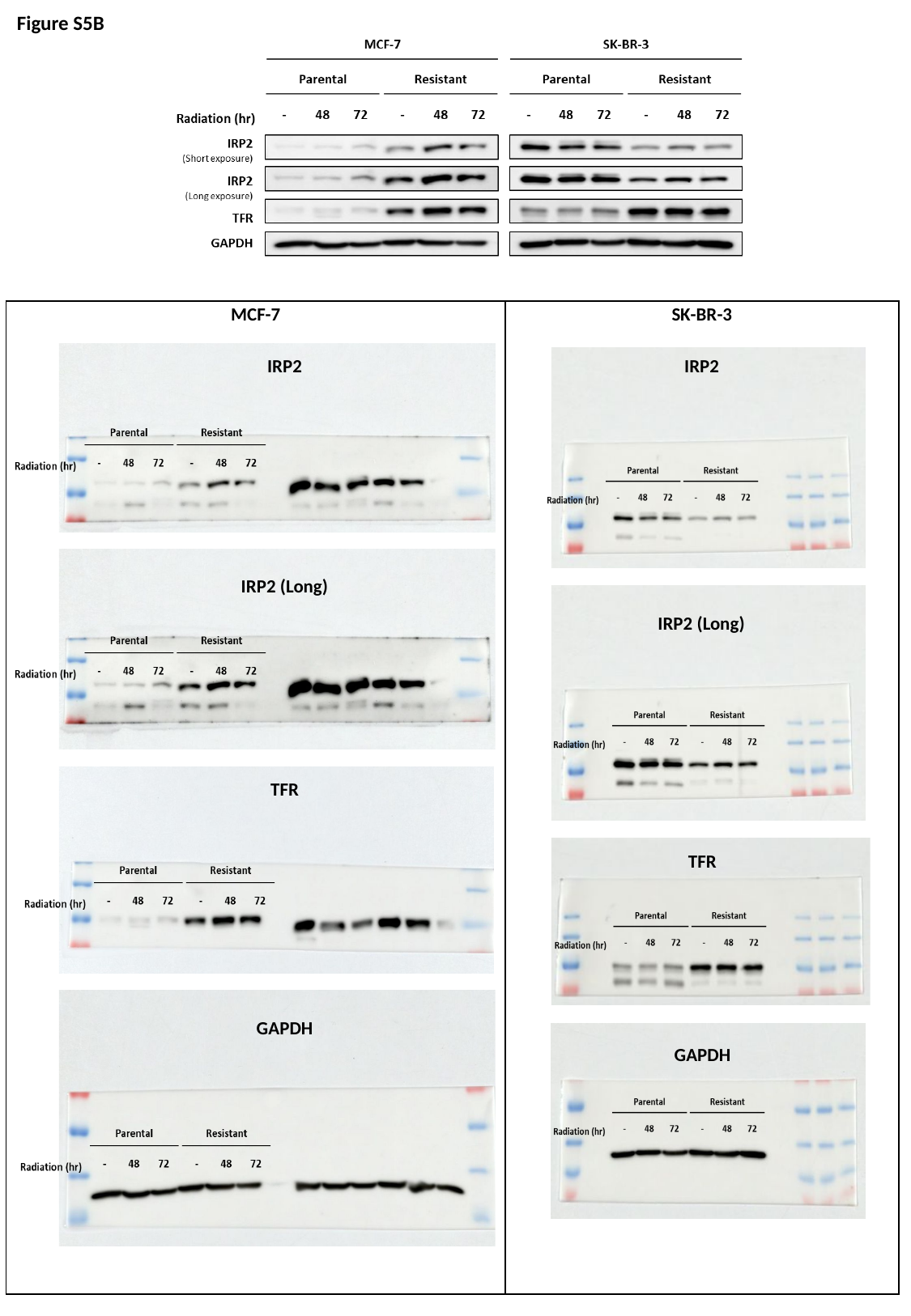

Figure S5B
| MCF-7 |
| --- |
| SK-BR-3 |
| --- |
| IRP2 |
| --- |
| IRP2 |
| --- |
| IRP2 (Long) |
| --- |
| IRP2 (Long) |
| --- |
| TFR |
| --- |
| TFR |
| --- |
| GAPDH |
| --- |
| GAPDH |
| --- |

## Slide 7
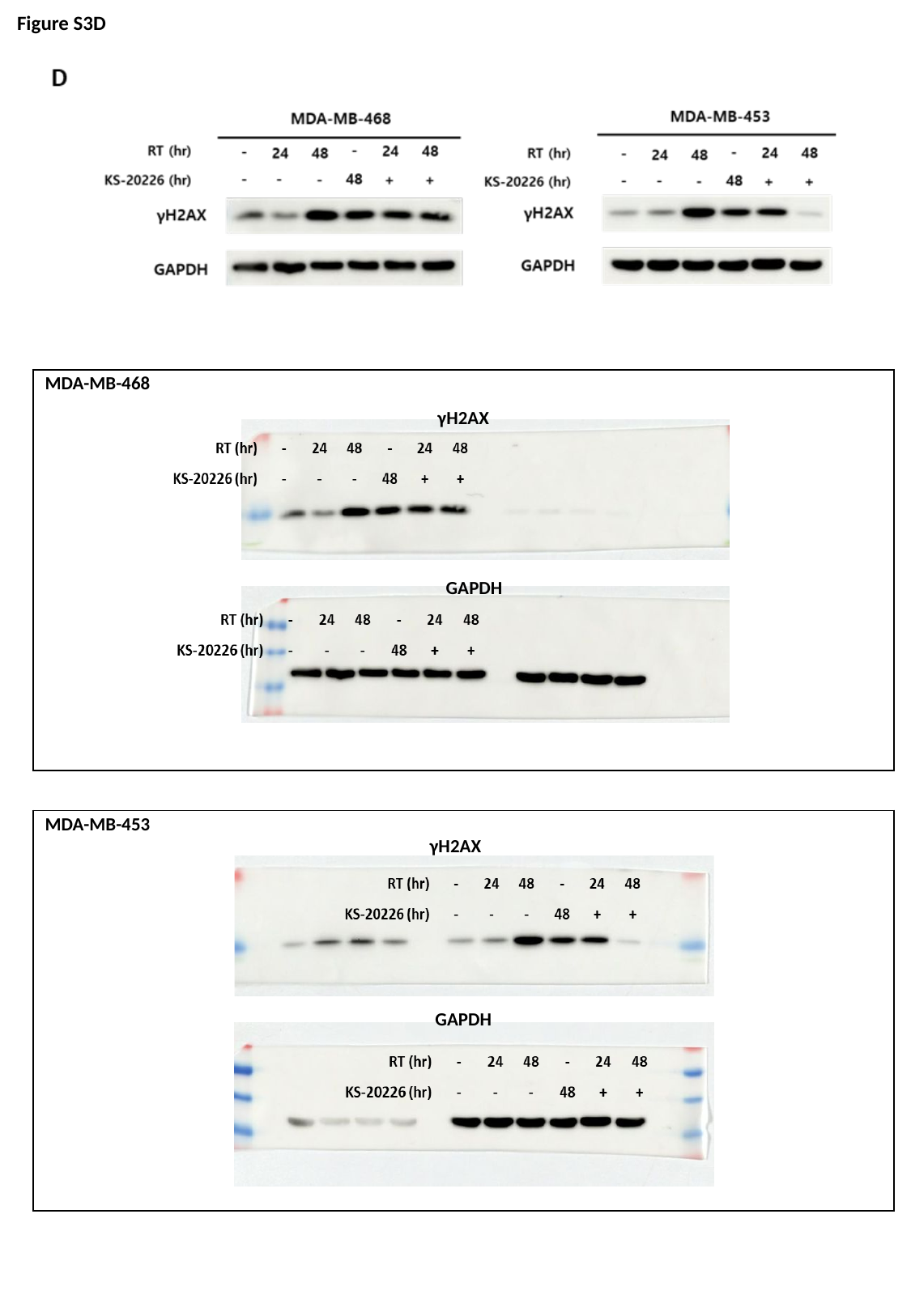

Figure S3D
| MDA-MB-468 |
| --- |
| γH2AX |
| --- |
| GAPDH |
| --- |
| MDA-MB-453 |
| --- |
| γH2AX |
| --- |
| GAPDH |
| --- |

## Slide 8
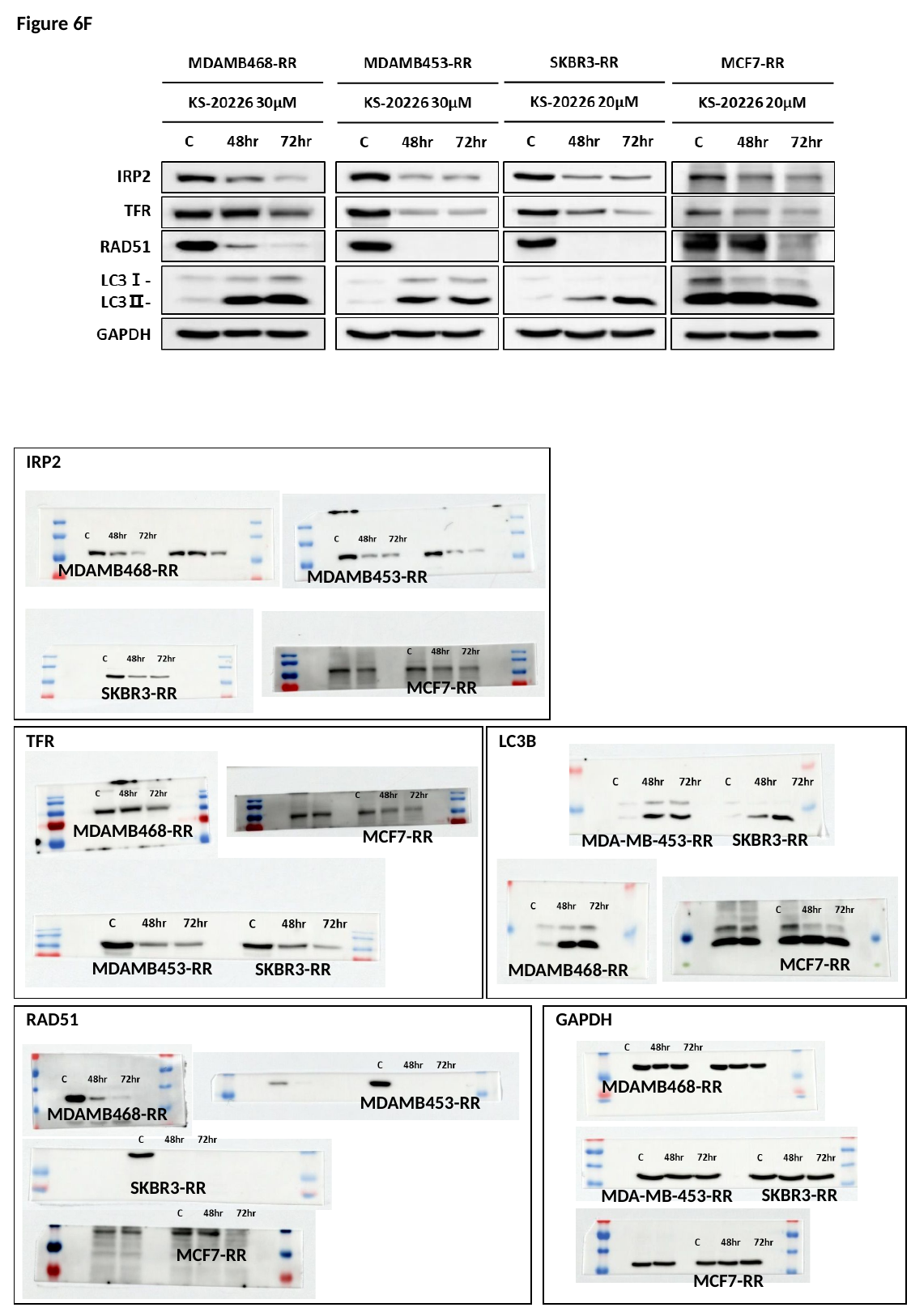

Figure 6F
| IRP2 |
| --- |
| MDAMB468-RR |
| --- |
| MDAMB453-RR |
| --- |
| MCF7-RR |
| --- |
| SKBR3-RR |
| --- |
| LC3B |
| --- |
| TFR |
| --- |
| MDAMB468-RR |
| --- |
| MCF7-RR |
| --- |
| SKBR3-RR |
| --- |
| MDA-MB-453-RR |
| --- |
| MCF7-RR |
| --- |
| MDAMB453-RR |
| --- |
| SKBR3-RR |
| --- |
| MDAMB468-RR |
| --- |
| RAD51 |
| --- |
| GAPDH |
| --- |
| MDAMB468-RR |
| --- |
| MDAMB453-RR |
| --- |
| MDAMB468-RR |
| --- |
| SKBR3-RR |
| --- |
| SKBR3-RR |
| --- |
| MDA-MB-453-RR |
| --- |
| MCF7-RR |
| --- |
| MCF7-RR |
| --- |

## Slide 9
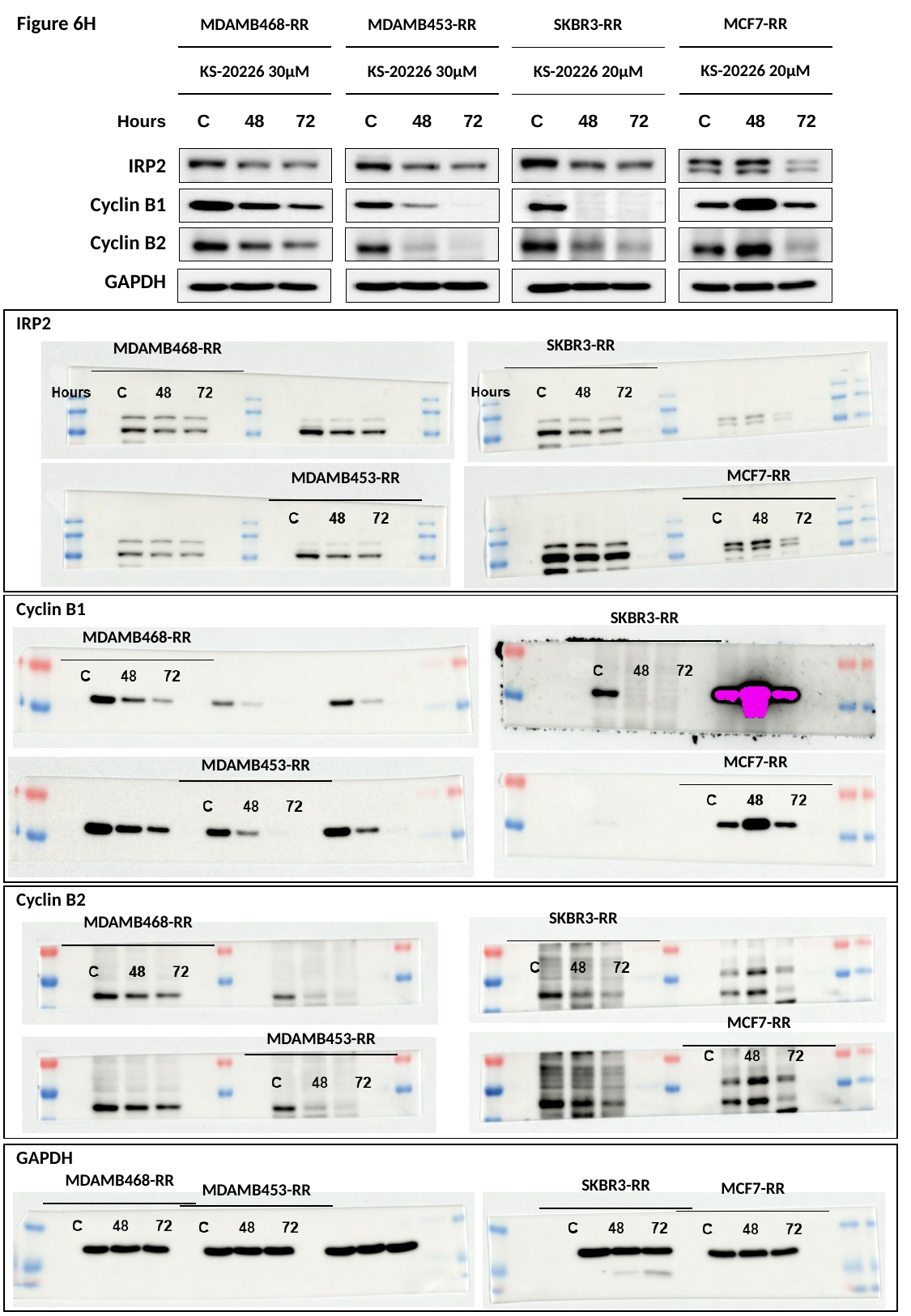

| MCF7-RR |
| --- |
Figure 6H
| MDAMB468-RR |
| --- |
| MDAMB453-RR |
| --- |
| SKBR3-RR |
| --- |
| KS-20226 20μM |
| --- |
| KS-20226 30μM |
| --- |
| KS-20226 30μM |
| --- |
| KS-20226 20μM |
| --- |
| Hours |
| --- |
| C | 48 | 72 |
| --- | --- | --- |
| C | 48 | 72 |
| --- | --- | --- |
| C | 48 | 72 |
| --- | --- | --- |
| C | 48 | 72 |
| --- | --- | --- |
| IRP2 |
| --- |
| Cyclin B1 |
| Cyclin B2 |
| GAPDH |
| IRP2 |
| --- |
| SKBR3-RR |
| --- |
| MDAMB468-RR |
| --- |
| MCF7-RR |
| --- |
| MDAMB453-RR |
| --- |
| Cyclin B1 |
| --- |
| SKBR3-RR |
| --- |
| MDAMB468-RR |
| --- |
| MCF7-RR |
| --- |
| MDAMB453-RR |
| --- |
| Cyclin B2 |
| --- |
| SKBR3-RR |
| --- |
| MDAMB468-RR |
| --- |
| MCF7-RR |
| --- |
| MDAMB453-RR |
| --- |
| GAPDH |
| --- |
| MDAMB468-RR |
| --- |
| SKBR3-RR |
| --- |
| MCF7-RR |
| --- |
| MDAMB453-RR |
| --- |
